# Enterprise-led internet healthcare provision in China: insights from a leading platform

**DOI:** 10.3389/fdgth.2025.1491183

**Published:** 2025-03-03

**Authors:** Li Wang, Dan Liang, Hengqian HuangFu, Changwen Ke, Shaolong Wu, Yingsi Lai

**Affiliations:** ^1^ Department of Medical Statistics, School of Public Health, Sun Yat-sen University, Guangzhou, China; ^2^ Department of Immunology and Microbiology, College of Life Science and Technology, Jinan University, Guangzhou, China; ^3^ Guangdong Provincial Center for Disease Control and Prevention, Guangdong Workstation for Emerging Infectious Disease Control and Prevention, Guangzhou, China; ^4^ Department of Public Administration, School of Government, Sun Yat-sen University, Guangzhou, China; ^5^ Sun Yat-sen Global Health Institute, Institute of State Governance, Sun Yat-sen University, Guangzhou, China; ^6^ Research Center of Health Informatics, Sun Yat-sen University, Guangzhou, China; ^7^ Guangdong Key Laboratory of Health Informatics, Guangzhou, China; ^8^ Guangzhou Joint Research Center for Disease Surveillance, Early Warning, and Risk Assessment, Guangzhou, China

**Keywords:** internet healthcare, internet healthcare platform, internet hospital, telemedicine, telehealth

## Abstract

**Background:**

China's healthcare resources are limited and unevenly distributed, with a notable urban-rural gap. Enterprise-led internet healthcare platforms have become an important solution for optimizing resource allocation, improving accessibility, and enhancing efficiency in mainland China. However, detailed analysis of their online consultation services from both healthcare provider and patient perspectives is still lacking.

**Objective:**

The online consultation data of an enterprise-led internet healthcare platform was depicted and analyzed to understand the temporal trend and current situation of enterprise-led internet healthcare development in mainland China, which provided insights for the further development of internet healthcare.

**Methods:**

We gathered information from an enterprise-led internet healthcare platform (i.e., Good Doctor Online) covering the period from January 2008 to December 2022, including the characteristics of doctors, healthcare institutions, and patients. Based on the above data, we sketched and analyzed the situation of online consultation services provided by the enterprise-led internet healthcare platform in mainland China.

**Results:**

A total of 149,890 doctors from 7,584 healthcare institutions provided 40,462,801 online consultations from January 2008 to December 2022. Doctors and healthcare institutions providing online consultation services were primarily distributed in the economically developed eastern and southern provinces of China. Doctors with intermediate (30.15%) and senior titles (58.12%) were the main providers of online consultations and most doctors were from tertiary hospitals (88.18%). The consultation price {*median* [interquartile range (*IQR*)]} was 49.00 (15.00, 100.00) RMB. The health issues with the highest consultation frequency included upper respiratory tract infections or fever (16.19%), gynecological disorders (11.98%), and skin diseases (8.65%), with variations in gender and age. The age distribution of patients showed two peaks in age groups <5 years and 20–39 years, with the median age (IQR) 29.00 (19.00–43.00) years.

**Conclusions:**

Enterprise-led internet healthcare platforms enhance access to care and reduce offline resource strain, especially during COVID-19. They mainly address non-urgent conditions but cannot fully replace in-person care. Policies should focus on increasing elderly participation, engaging senior doctors, optimizing male-oriented services, expanding access to underserved areas, standardizing pricing, and broadening insurance reimbursement coverage to improve equity and sustainability.

## 1 Introduction

Internet healthcare, as a new medical service model, uses internet technology as a carrier and integrates traditional medicine to improve regional healthcare by means of information technologies such as cloud computing, the Internet of Things, and big data (1, 2). Currently, internet healthcare in China is experiencing a rapid development. In order to guide the development of internet healthcare, the Chinese government has issued a series of regulations and guidelines in the past several years. In July 2015, the State Council of the People's Republic of China issued the *Guidance on Actively Promoting the Action of “Internet Plus”*. It proposed to develop internet-based healthcare services, encourage internet enterprises to cooperate with medical institutions for establishing medical network information platforms, reinforce the integration of regional healthcare resources, and actively apply the mobile internet to explore and provide health services, such as online appointments or consultations, electronic prescriptions, and drug delivery (3). It was the first time that the integration of the internet and healthcare was advocated at the national level. In 2018, the National Health Commission and the National Administration of Traditional Chinese Medicine of the People's Republic of China formulated three policies to provide detailed instructions on the admission criteria, practice rules, and supervision and management of internet medical institutions, and further regulate internet plus healthcare (4). In 2020, the National Healthcare Security Administration issued the *Guidance on Actively Promoting the Payment of Medical Insurance for “Internet Plus” Medical Services*, addressing a critical barrier to internet healthcare development by incorporating medical insurance coverage (5).

Depending on the different initiators, there are three main modes of internet healthcare service provision in mainland China: hospital-led, government-led, and enterprise-led internet healthcare platforms (6). The hospital-led mode extends traditional medical institutions' services (such as appointment, pre-clinic consultations, and post-clinic follow-ups) through internet tools. However, this model is limited by the lack of interoperability between different hospitals and inadequate scalability, which restricts its ability to effectively share resources and reduce healthcare costs (7). The government-led mode is typically initiated by the government and health administration departments, aiming to integrate regional medical institutions into a unified internet medical service cloud platform. This model primarily addresses public health needs but is often hindered by bureaucratic constraints and slower responsiveness to dynamic healthcare demands (6). In contrast, the enterprise-led mode, which establishes internet healthcare platforms by integrating hospitals, doctors, and patients, demonstrates significant advantages in scalability, resource allocation, and service diversification (6). This model, driven by market mechanisms, efficiently connects healthcare providers and users, making it the most predominant mode of internet healthcare in mainland China. Enterprise-led internet healthcare platforms leverage advanced technologies such as big data and artificial intelligence to optimize medical resource allocation, enhance patient access to high-quality healthcare, and improve service efficiency. These platforms are particularly effective in addressing the uneven geographical distribution of healthcare resources in China, where disparities between urban and rural areas remain significant (8–11). Giving these advantages, the enterprise-led model has demonstrated significant potential in tackling key challenges within the healthcare system, such as optimizing resource allocation, reducing cross-infections in hospitals, overcoming geographical constraints, and decreasing overall healthcare costs while improving efficiency (12–18). Therefore, our study focuses specifically on investigating the enterprise-led internet healthcare model to better understand its supply-demand characteristics and temporal development.

Existing studies had analyzed the characteristics of demanders (patients) and factors influencing their use of internet healthcare. For example, Gong et al. conducted an analysis of online consultation data from multiple internet hospitals across China during the COVID-19 pandemic. They found that the median age of individuals seeking online consultations is 28 years (IQR 22–33 years). Additionally, more than 90% of those seeking consultations exhibited symptoms associated with an COVID-19-like epidemic (13). Similarly, a study conducted a comparison between remote healthcare and traditional in-person visits, finding that the number of telemedicine consultations closely matches that of face-to-face encounters. Younger patients, residents of remote regions, and mothers with higher educational backgrounds demonstrate a higher inclination towards accepting telemedicine consultations (19). Sang et al. analyzed the utilization of internet hospitals among patients with pain and observed that younger, employed individuals, and those residing further from hospitals are more inclined to seek services from internet hospitals (20). The study by Cui et al. found that the main focus of remote consultations is on chronic diseases (21). An investigation has been conducted into the usage of telemedicine among rural dwellers in Guangdong Province, China, along with factors affecting its utilization. Major impediments to the acceptance of telemedicine among rural residents encompass deficiencies in knowledge, trust, demand, as well as insufficient material and social support (22).

There were also studies that described and analyzed the characteristics and roles of the supplier (such as internet hospitals) in internet healthcare. For example, Li et al.'s study found that during the early stages of COVID-19, internet hospitals can play a crucial role in disseminating timely and rapid information regarding the prevention and control of COVID-19. This aided in alleviating psychological burdens among consultees and enhances disease awareness (23). A study found that by enhancing healthcare capacity, quality, and efficiency in regions lacking adequate services and addressing the unequal allocation of healthcare resources, telemedicine offered a solution to the challenges of accessibility and high expenses in China's healthcare service (12).

However, while these studies provide valuable insights into specific aspects of internet healthcare, such as patient demographics, rural healthcare challenges, or the general benefits of telemedicine, they often examine these issues in isolation. A more comprehensive and systematic analysis of enterprise-led internet healthcare, particularly from the dual perspectives of supply and demand, remains largely absent in existing research. To address this gap, it is crucial to explore the supply-demand characteristics and temporal changes of enterprise-led internet healthcare, which plays a pivotal role in China's healthcare system by bridging resource disparities and enhancing service efficiency.

Good Doctor Online, established in 2006, exemplifies the enterprise-led internet healthcare model and serves as one of China's leading platforms in this field. As of July 2023, approximately 270,000 doctors have authentically registered on the platform, offering online medical services directly to patients. Among these active doctors, over 70% are affiliated with three-A hospitals, contributing to the platform's high medical service authority. Users can conveniently connect with doctors through various platforms such as the Haodf Online app, PC website, mobile website, WeChat official account, and WeChat mini-program, addressing a wide range of medical issues, through a one-step solution (24). Given its significant influence and wide-ranging impact in the field, Good Doctor Online was chosen as a representative example of an enterprise-led internet healthcare platform for this study. By analyzing various aspects of online consultation from both the supply and demand perspectives, our objective is to explore the current state of enterprise-led internet healthcare development in China and provide actionable suggestions for its further improvement.

## 2 Methods

### 2.1 Data collection

From September 10, 2023 to October 10, 2023, we collected the online consultation information from the enterprise-led internet healthcare platform, Good Doctor Online (haodf.com), in China covering the period from January 2008 to December 2022. The dataset consists of a total of 40,462,801 online consultations conducted by 149,890 doctors affiliated with 7,5 healthcare institutions across 31 provinces in mainland China. The data acquisition process is illustrated in the diagram shown in Supplementary Figure S1. The dataset includes detailed records of online consultations, including doctor characteristics (e.g., professional title, affiliated healthcare institution, and specialty), patient demographics (e.g., age and gender), consultation pricing, and health issues addressed during consultations (Supplementary Appendix 1). We also obtained the geographical locations of the healthcare institutions using the Amap API (https://restapi.amap.com/v3/geocode/geo?parameters) to determine the province of each healthcare institution. The doctors and healthcare institutions in the above internet healthcare platform were widely distributed in each province in mainland China, and the platform included almost all departments of offline hospitals. To provide additional context, we obtained the annual Per Capita Regional Gross Domestic Product (PCRGDP) data for 31 provinces in mainland China from the National Bureau of Statistics (data.stats.gov.cn) for the years 2008–2022. This economic data was incorporated into our analysis to examine potential relationships between regional economic development and online consultation pricing trends.

### 2.2 Data processing and analysis

To facilitate the subsequent analysis of the data, we further organized and classified the doctors' professional titles, department names, healthcare institutions, and common health issues in online consultations. The specific processing method is as follows: (i) Doctors' professional titles were classified into three levels: primary, intermediate, and senior, according to the title description. (ii) As for the problem of non-uniform naming of departments, we re-grouped departments into 20 categories according to the original department names, department profiles, and the scope of doctors practicing in the departments. (iii) We divided the healthcare institutions into hospitals and other healthcare institutions. Hospitals were further categorized into primary, secondary, tertiary, and unclassified hospitals according to the level of medical services provided, facility and equipment standards, medical technology capabilities, and the scope of responsibilities. (iv) We identified the 24 most frequently consulted health issues through a systematic analysis of online consultation records. First, we extracted all available disease diagnoses and consultation descriptions from the dataset and ranked them based on the total number of consultations. The top 24 most commonly consulted health issues were selected based on consultation volume, reflecting the most prevalent concerns among online healthcare users. We then organized them into binary classification variables for analysis. To improve clarity and consistency, similar conditions were grouped under broader categories where appropriate (e.g., rhinitis and sinusitis were classified together, as were hypertension, hyperglycemia, and hyperlipidemia). The final classification included a mix of acute and chronic illnesses, preventive healthcare services, and specialized medical concerns, covering a wide spectrum of internal medicine, dermatology, gynecology, cardiology, ophthalmology, and other major specialties. This categorization was structured based on consultation patterns observed in the dataset and aligned with the nature of enterprise-led internet healthcare services. (v) For a small proportion of records (0.007%) with missing consultation prices, the median online consultation prices of doctors with the same title in the same department were used to fill.

Considering that the data distribution does not follow a normal distribution, the *median* (*IQR*) was used to describe the number of consultations, consultation price, and patient age. For the categorical data, frequency, and percentage were utilized for description. We also counted the number of active doctors, where active doctor is defined as those who has conducted at least one online consultation during a specific period (e.g., one year). To analyze the relationship between PCRGDP and consultation prices, we calculated the weighted correlation coefficient [its 95% confidence interval (CI) was calculated through the bootstrap method] between them and fitted a weighted loess regression. The weights were based on the annual number of online consultations in each province. Data acquisition, cleaning, analysis, and a majority of data visualization were conducted in R (version 4.2.3). The map plotting was accomplished using ArcGIS (version 10.8).

## 3 Results

### 3.1 Overview

Up to December 2022, a total of 149,890 doctors from 7,584 healthcare institutions provided 40,462,801 online consultations on the platform of Good Doctor Online. The median consultation price was 49.00 (IQR 15.00–100.00) RMB, and the median patient age was 29.00 (IQR 19.00–43.00) years (Table 1, Supplementary Table S1, and Table S2).

**Table 1:** Characteristics of the internet healthcare provision in mainland China.

Characteristics	Number of doctors, *n* (%)	Total number of consultations, *n* (%)	Number of consultations provided per active doctor, median (*IQR*)	Consultation price, median (*IQR*) RMB
Professional title				
Primary title	17573 (11.72)	1,361,385 (3.36)	8.00 (2.00, 35.00)	9.00 (0.00, 20.00)
Intermediate title	45,194 (30.15)	8,242,597 (20.37)	19.00 (4.00, 111.00)	29.00 (9.00, 50.00)
Senior title	87,123 (58.12)	30,858,819 (76.26)	52.00 (7.00, 349.00)	50.00 (25.00, 100.00)
Healthcare institution				
Hospital	147,693 (98.53)	39,514,025 (97.66)	28.00 (5.00, 201.00)	49.00 (15.00, 100.00)
Primary hospital	802 (0.54)	174,648 (0.43)	16.00 (3.00, 117.50)	9.00 (0.00, 30.00)
Secondary hospital	14,600 (9.74)	2,089,403 (5.16)	10.00 (2.00, 59.00)	9.00 (6.00, 25.00)
Tertiary hospital	132,178 (88.18)	37,217,746 (91.98)	32.00 (5.00, 224.00)	50.00 (19.00, 100.00)
Unclassified hospital	113 (0.08)	32,228 (0.08)	55.00 (14.00, 275.00)	50.00 (30.00, 100.00)
Other healthcare institution	2,197 (1.47)	948,776 (2.34)	59.00 (8.00, 418.00)	30.00 (9.00, 99.00)
Total	149,890 (100.00)	40,462,801 (100.00)	29.00 (5.00, 203.00)	49.00 (15.00, 100.00)

### 3.2 Doctors providing online consultations

**Figure 1:**
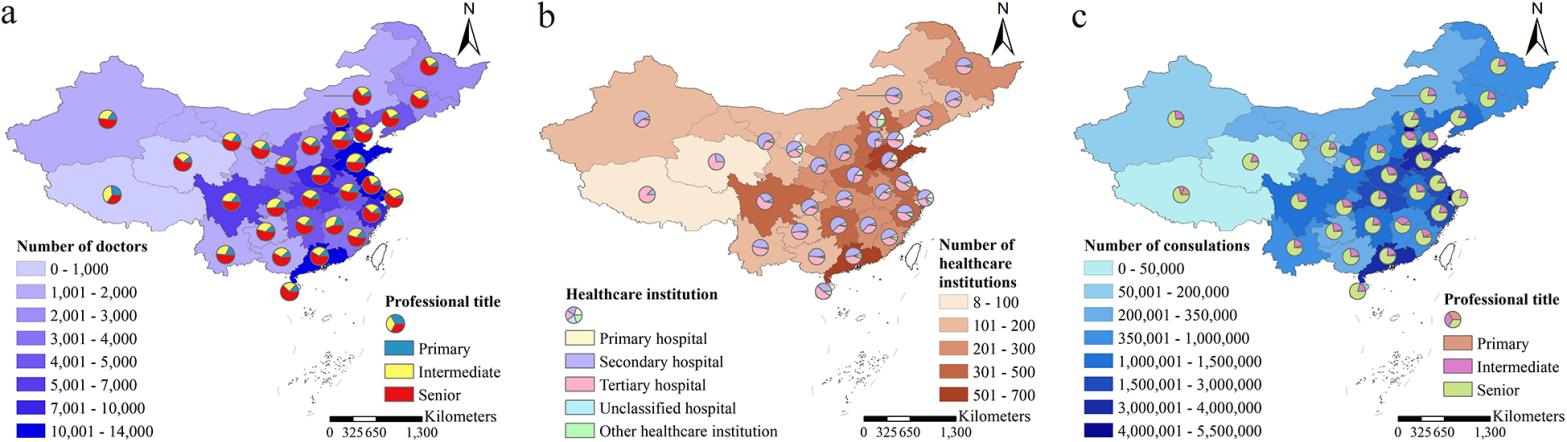
The distribution of doctors, healthcare institutions, and the number of consultations. **(a)** The distribution of doctors providing online consultations, including both the total number of doctors and the proportion categorized by their professional titles, was presented. **(b)** The distribution of healthcare institutions affiliated with doctors providing online consultations, including the total number of healthcare institutions and the proportion of each category, was provided. **(c)** The distribution of the number of doctors providing online consultations, including the total number of consultations and the proportion of consultations provided by doctors of different professional titles.

The doctors providing online consultations and their affiliated healthcare institutions were primarily concentrated in the eastern and southern regions of China (Figures 1a,b). The top five provinces with the highest number and percentage of online consultation doctors were Guangdong [13,684 (9.13%)], Shandong [13,508 (9.01%)], Beijing [13,069 (8.72%)], Shanghai [12,958 (8.65%)], and Jiangsu [10,134 (6.76%)] (Figure 1c, Supplementary Figure S2c and Table S2).

Doctors with senior professional titles were the predominant group providing online consultations, with a totaling of 87,123 (58.12%), followed by 45,194 (30.15%) with intermediate titles, and 17,573 (11.72%) with primary titles (Table 1 and Supplementary Figure S2a). The majority of these doctors were affiliated with hospitals (147,693 out of 149,890, 98.53%), and as the hospital grade increases, the number of doctors also tends to rise. The distribution of doctors in primary, secondary, and tertiary hospitals was 802 (0.54%), 14,600 (9.74%), and 132,178 (88.18%), respectively (Table 1 and Supplementary Figure S2a). During the period from 2008 to 2022, the number of active doctors showed a general upward trend, with a predominant presence of senior title doctors among the active practitioners (Supplementary Figure S2b and Table S2). The top five departments with the highest number of online consultation doctors were the Traditional Chinese Medicine Department, Otorhinolaryngology Department, Pediatrics Department, General Surgery Department, and Obstetrics and Gynecology Department (Supplementary Figure S2d and Table S3). Among these departments, the proportion of senior title doctors exceeded 50% (Supplementary Table S3). Taking into account both the doctors' titles and the healthcare institutions they affiliated to, the majority doctors were with senior title from tertiary hospitals (79,640; 53.13%), followed by doctors with intermediate title from the same category of hospitals (38,319; 25.56%) (Supplementary Figure S2a and Table S4).

### 3.3 Total number of consultations

**Figure 2:**
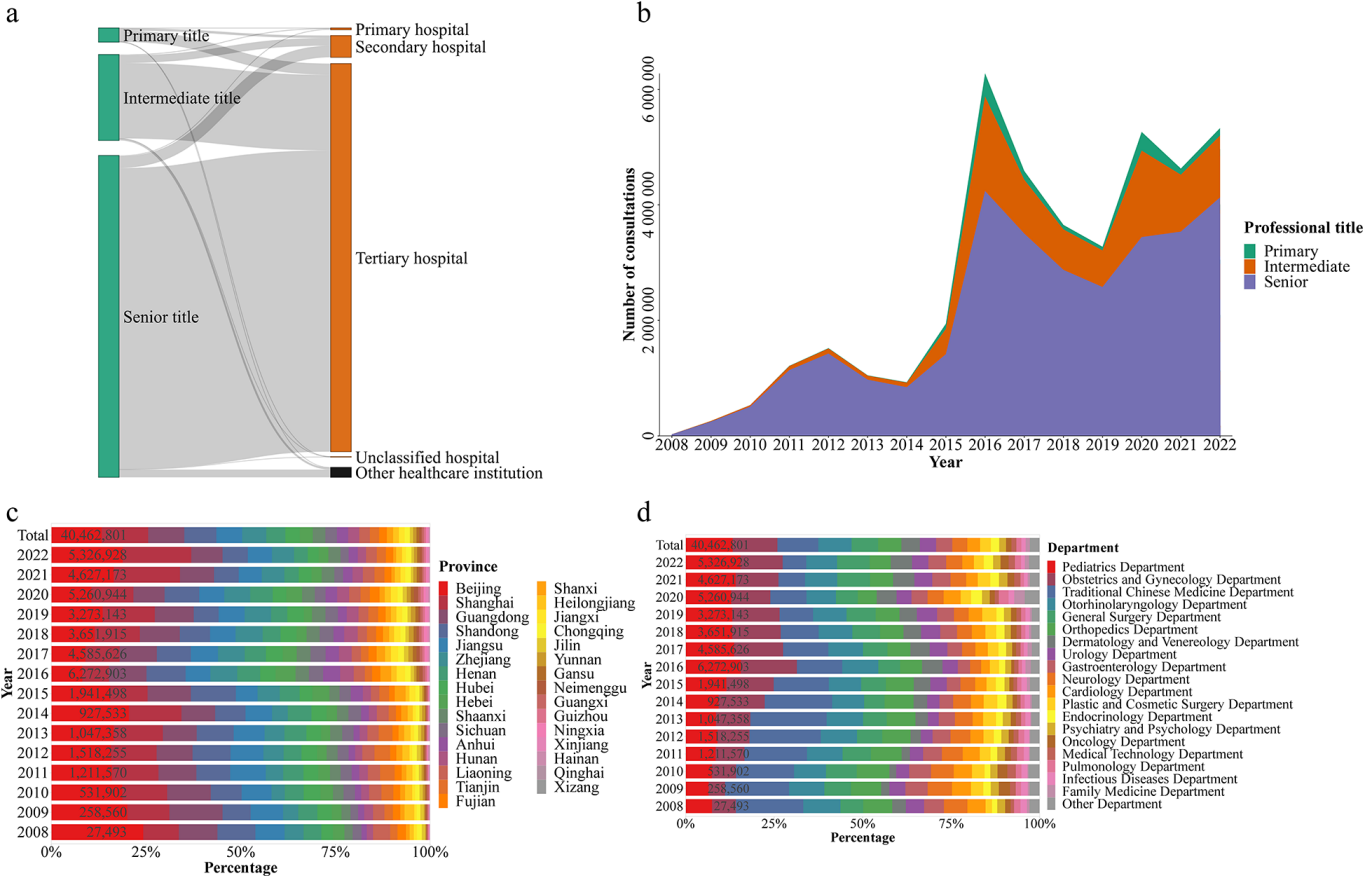
Characteristics of the number of online consultations. The composition of the number of online consultations, grouped by doctors’ professional titles and the healthcare institutions with which the doctors were affiliated, was provided **(a)** The number of online consultations, grouped by professional title **(b)**, province **(c)**, and department **(d)**, for each year.

Similar to the distribution of doctors, those with senior professional titles from tertiary hospitals provided the highest number of online consultations, with a total of 28,906,368 (71.44%), followed by those with intermediate titles from tertiary hospitals at 7,243,804 (17.90%) (Supplementary Table S4 and Figure 2a). During the period from 2008 to 2022, the number of online consultations showed an overall upward trend, with doctors holding senior professional titles consistently being the primary providers of online consultation services (Figure 2b and Supplementary Table S4). Similar to the distribution of doctors and healthcare institutions providing online consultation services, provinces with a higher number of consultations were predominantly located in the eastern and southern regions of China. The top five provinces with the highest total number of consultations were Beijing, Shanghai, Guangdong, Shandong, and Jiangsu (Figures 1c, 2c, and Supplementary Table S5). Additionally, the top five departments with the highest number of consultations were the Pediatrics Department, Obstetrics and Gynecology Department, Traditional Chinese Medicine Department, Otorhinolaryngology Department, and General Surgery Department (Figure 2d and Supplementary Table S5).

### 3.4 Number of consultations provided per active doctor

During the period from 2008 to 2022, the annual number of consultations provided per active doctor did not show a significant upward trend (Supplementary Figures S3a–c). However, with the higher the professional titles, the median number of consultations provided per active doctor gradually increased (Supplementary Figure S3a and Table S6). For doctors with primary, intermediate, and senior professional titles, the median number of consultations provided per doctor were 8.00 (IQR 2.00–35.00), 19.00 (IQR 4.00–111.00), and 52.00 (IQR 7.00–349.00), respectively (Table 1 and Supplementary Table S6).

### 3.5 Consultation price

**Figure 3:**
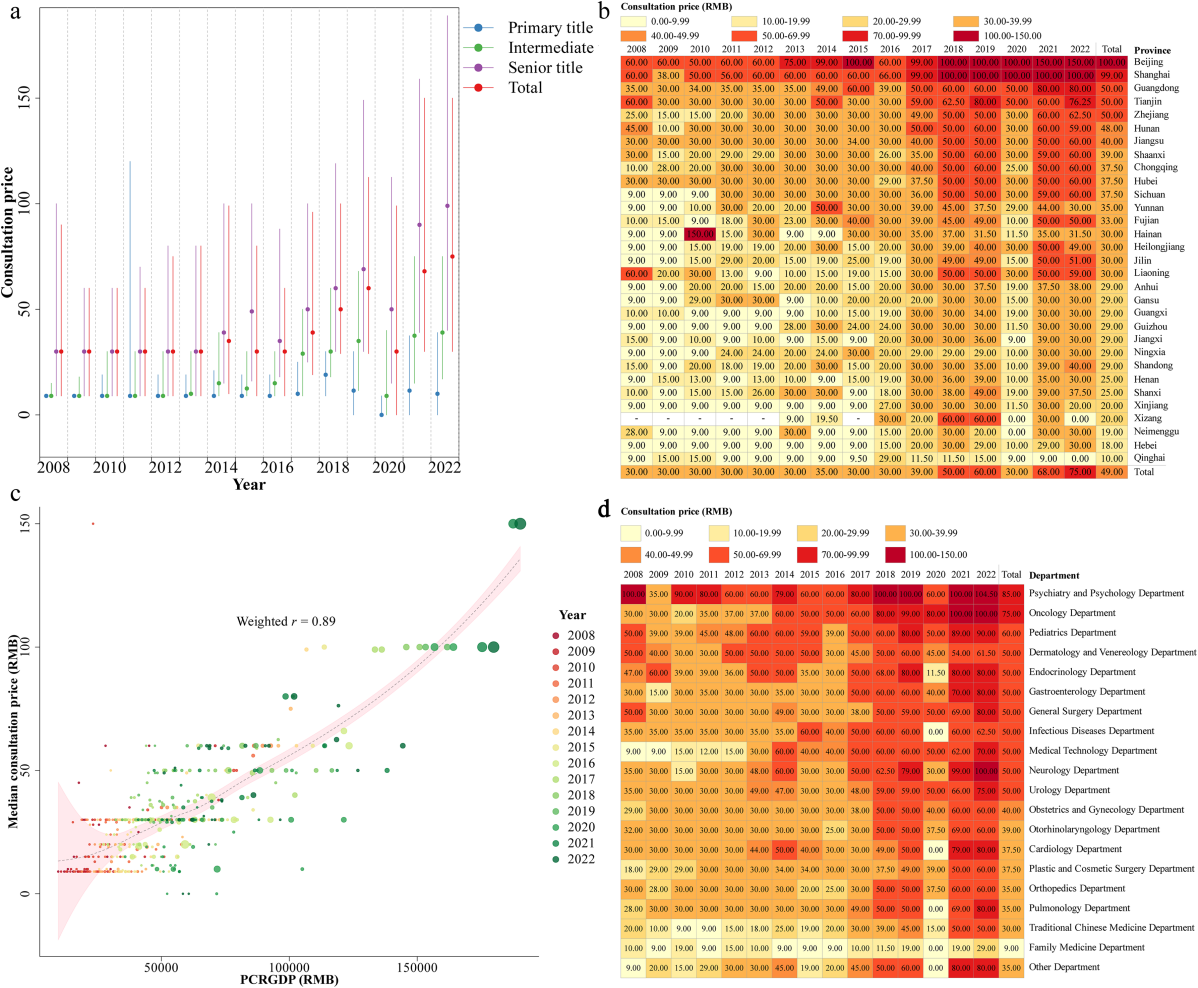
Characteristics of the median consultation price. The online median consultation price, grouped by professional title **(a)**, province **(b)** and department **(d)**, for each year. A scatter plot of the annual PCRGDP and median consultation prices for each province **(c)** A weighted (the weights were based on the annual number of consultations in each province) correlation coefficient between PCRGDP and consultation price was calculated. We also fitted a weighted loess regression for the relationship between PCRGDP and consultation prices, with weights consistent with that used in calculating weighted correlation coefficient. PCRGDP, Per Capita Regional Gross Domestic Product.

The higher the professional title of the doctor, the higher the consultation price. The median consultation prices for doctors with primary, intermediate, and senior professional titles were 9.00 (IQR 0.00–20.00) RMB, 29.00 (IQR 9.00–50.00) RMB, and 50.00 (IQR 25.00–100.00) RMB, respectively (Table 1). Overall, consultation prices showed an increasing trend from 2008 to 2022 (Figures 3a–d and Supplementary Table S7). During the period of the COVID-19 pandemic from 2019 to 2022, there was a significant increase in the provision of free consultations (with a consultation price of 0 RMB). The proportion of free consultations in 2020, 2021, and 2022 were 31.01%, 9.29%, and 10.13% respectively (Supplementary Figure S4). The consultation prices exhibited a strong correlation with the PCRGDP of each province, with a weighted correlation coefficient of 0.89 (95% CI 0.83–0.92) (Figure 3c). The top five provinces with the highest median consultation prices were Beijing [100.00 (IQR 50.00–200.00) RMB], Shanghai [99.00 (IQR 49.00–199.00) RMB], Guangdong [50.00 (IQR 26.00–100.00) RMB], Tianjin [50.00 (IQR 25.00–100.00) RMB], and Zhejiang [50.00 (IQR 20.00–100.00) RMB] (Supplementary Table S7). Additionally, there were variations in consultation prices across different departments, with a median price of 85.00 (IQR 30.00–200.00) RMB for Psychiatry and Psychology Department, 75.00 (IQR 30.00–199.00) RMB for Oncology Department, and 60.00 (IQR 30.00–119.00) RMB for Pediatrics Department ranking as the top three departments with higher consultation prices (Supplementary Table S7).

### 3.6 The age distribution of patients seeking online consultations

**Figure 4:**
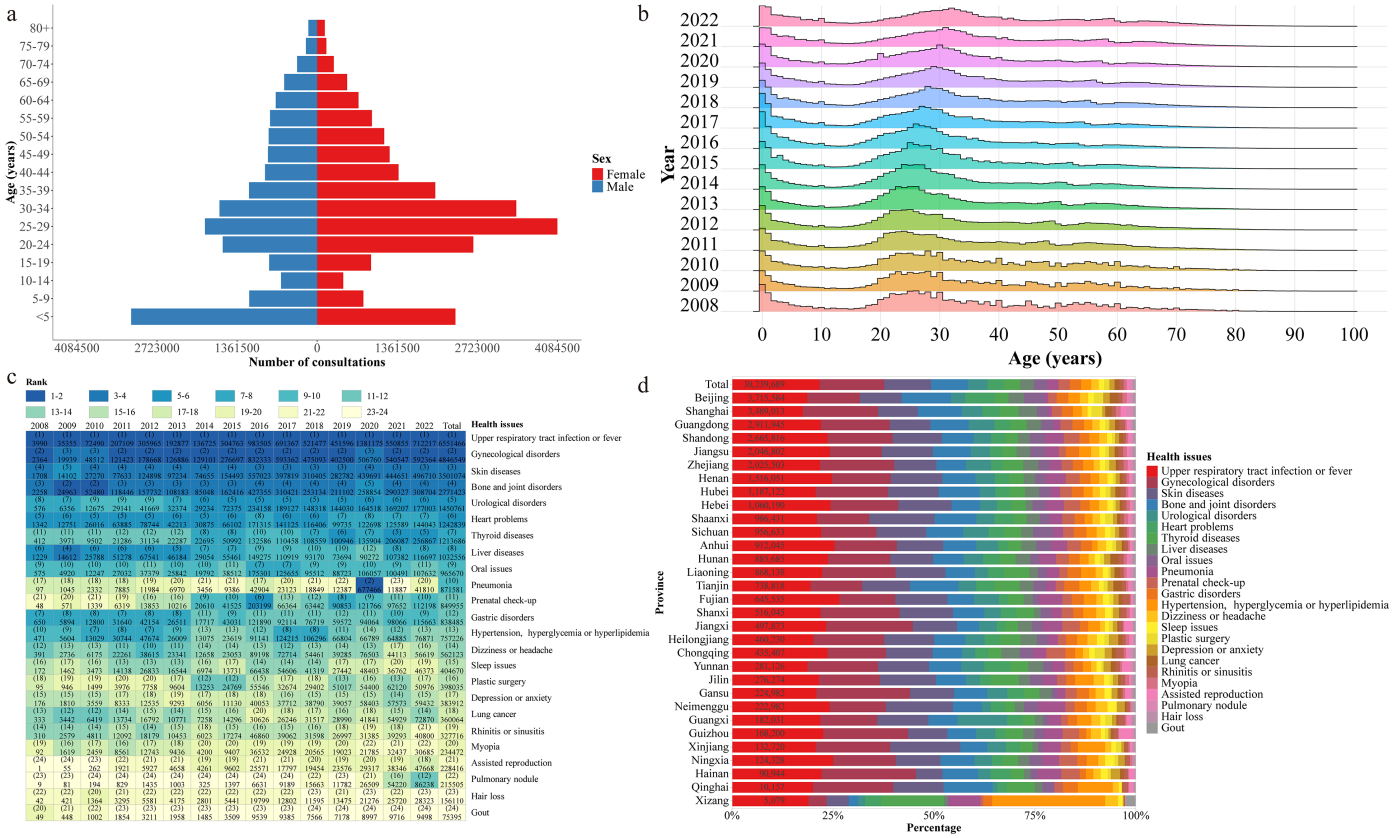
Characteristics of the age and primary health issues of patients seeking online consultations. **(a)** The age distribution of patient seeking online consultations, grouped by gender. **(b)** The age distribution of patient seeking online consultations, grouped by year. **(c)** The frequency ranking of patients’ main health issues, grouped by year. **(d)** The frequency and composition of main health issues in consultations, grouped by province.

The age distribution of online consultation patients remained relatively consistent each year, with median ages for males and females being 28.00 (IQR 10.00–45.00) and 30.00 (IQR 22.00–42.00) years, respectively (Supplementary Table S1). Online consultation patients were predominantly concentrated in the age groups < 5 years and 20–39 years (Figure 4a,b), accounting for a proportion of 13.62% and 45.63%, respectively. In contrast, the age group ≥ 60 years had fewer consultations, accounting for only 9.20% of the total (Supplementary Table S1). The number of online consultations by females consistently exceeded those of males each year (Supplementary Table S1). Females had a higher frequency of consultations compared to males across most age groups, with the difference being particularly pronounced in the 20–39 age group (Figure 4a). The age group with the highest number of male consultations was <5 years, while for females it was the 25–29 years age group (Figure 4a).

### 3.7 The primary health issues in online consultations

The primary health issues in each province showed little variation, with the top five health issues in consultations being upper respiratory tract infections or fever, gynecological disorders, skin diseases, bone and joint issues, and urological disorders (Figures 4c,d). For most years, the leading three health issues in consultations remained consistent as upper respiratory tract infections or fever, gynecological disorders, and skin diseases (Figure 4c, Table 2). Notably, with the outbreak of COVID-19 in 2020, pneumonia consultations experienced a significant surge, ranking as the second-highest health issue by consultation frequency (Figure 4c). The distribution of primary consultation health issues varied by gender and age. For females under 40 years old, the top three consultation diseases or symptoms were upper respiratory tract infections or fever, skin diseases, and gynecological disorders. After the age of 40 years, bone and joint issues and heart problems replaced skin diseases and gynecological disorders as the top three consultation health issues (Table 2). For males under 40 years old, the top three consulted diseases or symptoms were upper respiratory tract infections or fever, urological disorders, and skin diseases. For males aged 40 and above, bone and joint issues, heart problems, and liver disease gradually replaced urological disorders and skin diseases to become the prominently consulted health issues (Table 2).

**Table 2:** The number and proportions of consultations for the primary health issues in online consultations by different genders and age groups.

Health issue	<20 years, *n* (%)			20–39 years, *n* (%)			40–59 years, *n* (%)			≥60 years, *n* (%)			All ages, *n* (%)		
	Female	Male	Total	Female	Male	Total	Female	Male	Total	Female	Male	Total	Female	Male	Total
Assisted reproduction	1,477 (0.03)	266 (0.00)	1,743 (0.02)	194,412 (1.60)	13,356 (0.21)	207,768 (1.13)	16,988 (0.36)	1,790 (0.05)	18,778 (0.23)	72 (0.00)	55 (0.00)	127 (0.00)	212,949 (0.92)	15,467 (0.09)	228,416 (0.56)
Bone and joint disorders	244,529 (5.44)	272,047 (4.74)	516,576 (5.04)	503,406 (4.15)	587,146 (9.27)	1,090,552 (5.91)	438,293 (9.35)	347,047 (10.37)	785,340 (9.78)	225,018 (12.63)	153,937 (7.92)	378,955 (10.18)	1,411,246 (6.11)	1,360,177 (7.83)	2,771,423 (6.85)
Depression or anxiety	55,413 (1.23)	30,938 (0.54)	86,351 (0.84)	123,980 (1.02)	80,374 (1.27)	204,354 (1.11)	44,651 (0.95)	25,043 (0.75)	69,694 (0.87)	15,863 (0.89)	7,650 (0.39)	23,513 (0.63)	239,907 (1.04)	144,005 (0.83)	383,912 (0.95)
Dizziness or headache	37,248 (0.83)	38,869 (0.68)	76,117 (0.74)	144,775 (1.19)	111,807 (1.77)	256,582 (1.39)	98,395 (2.10)	63,461 (1.90)	161,856 (2.01)	39,235 (2.20)	28,333 (1.46)	67,568 (1.81)	319,653 (1.38)	242,470 (1.40)	562,123 (1.39)
Gastric disorders	52,292 (1.16)	61,769 (1.08)	114,061 (1.11)	162,083 (1.34)	150,023 (2.37)	312,106 (1.69)	130,729 (2.79)	132,158 (3.95)	262,887 (3.27)	58,307 (3.27)	91,124 (4.69)	149,431 (4.01)	403,411 (1.75)	435,074 (2.51)	838,485 (2.07)
Gout	605 (0.01)	4,347 (0.08)	4,952 (0.05)	2,995 (0.02)	34,929 (0.55)	37,924 (0.21)	2,078 (0.04)	21,809 (0.65)	23,887 (0.30)	1,508 (0.08)	7,124 (0.37)	8,632 (0.23)	7,186 (0.03)	68,209 (0.39)	75,395 (0.19)
Gynecological disorders	265,653 (5.91)	0 (0.00)	265,653 (2.59)	3,448,405 (28.42)	0 (0.00)	3,448,405 (18.68)	1,004,728 (21.43)	0 (0.00)	1,004,728 (12.51)	127,763 (7.17)	0 (0.00)	127,763 (3.43)	4,846,549 (20.98)	0 (0.00)	4,846,549 (11.98)
Hair loss	15,113 (0.34)	15,000 (0.26)	30,113 (0.29)	49,791 (0.41)	60,605 (0.96)	110,396 (0.60)	7,733 (0.16)	6,307 (0.19)	14,040 (0.17)	898 (0.05)	663 (0.03)	1,561 (0.04)	73,535 (0.32)	82,575 (0.48)	156,110 (0.39)
Heart problems	134,491 (2.99)	150,257 (2.62)	284,748 (2.78)	188,111 (1.55)	134,791 (2.13)	322,902 (1.75)	159,108 (3.39)	190,694 (5.70)	349,802 (4.35)	131,321 (7.37)	154,066 (7.93)	285,387 (7.66)	613,031 (2.65)	629,808 (3.63)	1,242,839 (3.07)
Hypertension, hyperglycemia or hyperlipidemia	11,356 (0.25)	14,770 (0.26)	26,126 (0.26)	107,539 (0.89)	102,263 (1.62)	209,802 (1.14)	142,739 (3.04)	181,239 (5.42)	323,978 (4.03)	102,314 (5.74)	95,006 (4.89)	197,320 (5.30)	363,948 (1.58)	393,278 (2.26)	757,226 (1.87)
Liver diseases	58,991 (1.31)	82,240 (1.43)	141,231 (1.38)	196,292 (1.62)	222,553 (3.52)	418,845 (2.27)	123,976 (2.64)	209,545 (6.26)	333,521 (4.15)	51,606 (2.90)	87,353 (4.50)	138,959 (3.73)	430,865 (1.87)	601,691 (3.47)	1,032,556 (2.55)
Lung cancer	1,194 (0.03)	1,758 (0.03)	2,952 (0.03)	11,809 (0.10)	9,408 (0.15)	21,217 (0.11)	68,655 (1.46)	84,793 (2.53)	153,448 (1.91)	63,832 (3.58)	118,615 (6.11)	182,447 (4.90)	145,490 (0.63)	214,574 (1.24)	360,064 (0.89)
Myopia	50,980 (1.13)	63,059 (1.10)	114,039 (1.11)	61,596 (0.51)	37,848 (0.60)	99,444 (0.54)	9,336 (0.20)	6,688 (0.20)	16,024 (0.20)	2,966 (0.17)	1,999 (0.10)	4,965 (0.13)	124,878 (0.54)	109,594 (0.63)	234,472 (0.58)
Oral issues	145,069 (3.23)	136,664 (2.38)	281,733 (2.75)	362,596 (2.99)	175,953 (2.78)	538,549 (2.92)	63,221 (1.35)	47,681 (1.43)	110,902 (1.38)	18,887 (1.06)	15,599 (0.80)	34,486 (0.93)	589,773 (2.55)	375,897 (2.16)	965,670 (2.39)
Plastic surgery	47,795 (1.06)	26,926 (0.47)	74,721 (0.73)	260,993 (2.15)	33,711 (0.53)	294,704 (1.60)	23,762 (0.51)	3,061 (0.09)	26,823 (0.33)	1,272 (0.07)	515 (0.03)	1,787 (0.05)	333,822 (1.45)	64,213 (0.37)	398,035 (0.98)
Pneumonia	112,321 (2.50)	137,416 (2.39)	249,737 (2.44)	228,039 (1.88)	178,945 (2.83)	406,984 (2.20)	89,361 (1.91)	74,806 (2.24)	164,167 (2.04)	22,982 (1.29)	27,711 (1.43)	50,693 (1.36)	452,703 (1.96)	418,878 (2.41)	871,581 (2.15)
Prenatal check-up	20,685 (0.46)	0 (0.00)	20,685 (0.20)	807,581 (6.66)	0 (0.00)	807,581 (4.37)	21,416 (0.46)	0 (0.00)	21,416 (0.27)	273 (0.02)	0 (0.00)	273 (0.01)	849,955 (3.68)	0 (0.00)	849,955 (2.10)
Pulmonary nodule	986 (0.02)	1,058 (0.02)	2,044 (0.02)	37,289 (0.31)	28,698 (0.45)	65,987 (0.36)	61,366 (1.31)	38,757 (1.16)	100,123 (1.25)	26,265 (1.47)	21,086 (1.09)	47,351 (1.27)	125,906 (0.55)	89,599 (0.52)	215,505 (0.53)
Rhinitis or sinusitis	62,084 (1.38)	115,501 (2.01)	177,585 (1.73)	54,917 (0.45)	56,007 (0.88)	110,924 (0.60)	17,733 (0.38)	15,920 (0.48)	33,653 (0.42)	2,851 (0.16)	2,703 (0.14)	5,554 (0.15)	137,585 (0.60)	190,131 (1.10)	327,716 (0.81)
Skin diseases	523,871 (11.65)	532,236 (9.27)	1,056,107 (10.31)	1,239,044 (10.21)	654,071 (10.33)	1,893,115 (10.25)	240,477 (5.13)	167,381 (5.00)	407,858 (5.08)	67,031 (3.76)	76,963 (3.96)	143,994 (3.87)	2,070,423 (8.96)	1,430,651 (8.24)	3,501,074 (8.65)
Sleep issues	50,011 (1.11)	61,309 (1.07)	111,320 (1.09)	91,779 (0.76)	74,319 (1.17)	166,098 (0.90)	58,766 (1.25)	37,033 (1.11)	95,799 (1.19)	18,778 (1.05)	12,675 (0.65)	31,453 (0.84)	219,334 (0.95)	185,336 (1.07)	404,670 (1.00)
Thyroid diseases	50,857 (1.13)	29,775 (0.52)	80,632 (0.79)	532,684 (4.39)	111,059 (1.75)	643,743 (3.49)	322,955 (6.89)	85,416 (2.55)	408,371 (5.08)	60,572 (3.40)	20,368 (1.05)	80,940 (2.17)	967,068 (4.19)	246,618 (1.42)	1,213,686 (3.00)
Upper respiratory tract infection or fever	797,850 (17.74)	1,044,882 (18.19)	1,842,732 (17.99)	1,765,090 (14.55)	1,245,872 (19.68)	3,010,962 (16.31)	727,442 (15.52)	524,367 (15.68)	1,251,809 (15.58)	240,115 (13.48)	205,848 (10.60)	445,963 (11.98)	3,530,497 (15.28)	3,020,969 (17.40)	6,551,466 (16.19)
Urological disorders	0 (0.00)	342,575 (5.96)	342,575 (3.35)	0 (0.00)	851,656 (13.45)	851,656 (4.61)	0 (0.00)	130,965 (3.92)	130,965 (1.63)	0 (0.00)	125,565 (6.46)	125,565 (3.37)	0 (0.00)	1,450,761 (8.36)	1,450,761 (3.59)

## 4 Discussion

### 4.1 Summary of findings

This study provides a comprehensive analysis of enterprise-led internet healthcare in China, integrating both supply- and demand-side perspectives based on a longitudinal dataset spanning 15 years (2008–2022). Our research identifies key trends in online consultations, including the predominance of senior doctors from tertiary hospitals, variations in consultation pricing, and differences in patient engagement across gender and age groups. While previous studies have typically focused on either the characteristics of online healthcare patients or the role of healthcare providers, our study bridges these perspectives to offer a more holistic understanding of enterprise-led platforms in China. Additionally, we extend the current literature by incorporating regional economic indicators (PCRGDP) to contextualize variations in consultation pricing across provinces. By demonstrating how enterprise-led internet healthcare models contribute to the optimization of medical resource distribution, the reduction of healthcare disparities, and the expansion of access to high-quality care, our findings provide valuable insights for both academic research and policy development.

### 4.2 Regional distribution of online healthcare services and policy interventions

Our study suggested that most of the doctors and healthcare institutions providing online consultation services in mainland China were currently concentrated in the economically developed eastern and southern regions, which was similar to the distribution of offline healthcare resources (25). However, this uneven distribution suggests a need for targeted policy interventions to promote internet healthcare adoption in central and western regions. Policymakers could introduce infrastructure investment, financial incentives, and digital healthcare training programs to encourage expansion in underserved areas, reducing disparities in medical resource distribution. In traditional offline healthcare, restricted by the economy, distance, and time, it was difficult for patients in remote areas in central and western regions to obtain healthcare resources from high-grade hospitals, while internet healthcare provides a possible way to obtain high quality healthcare services in developed regions, thus saving transportation and time costs (26).

### 4.3 The role of senior doctors and limitations of online consultations

Online consultation services were mainly provided by doctors with intermediate and senior titles from tertiary hospitals. This may be related to the fact that internet healthcare platforms expect to attract patients through high-quality medical resources. In addition, as patients were free to choose doctors, and many of them did not have confidence in primary healthcare providers and preferred to go to high-grade hospitals (18). To enhance the accessibility and credibility of online healthcare, policymakers should consider strategies to encourage more senior specialists to engage in online consultations while also strengthening online training programs for primary care providers. These measures would improve patient trust in lower-tier medical institutions and help distribute online consultations more evenly across different levels of the healthcare system.

Online consultations often presented limitations in diagnosis and treatment. When doctors diagnosed on internet healthcare platform, clear judgment of the disease was necessary for prescribing or offline referral. However, those often relied on laboratory and imaging tests from offline hospitals. Additionally, incomplete online healthcare procedures may also affect doctors' diagnosis of diseases. This determined to a certain extent that the main type of online healthcare service was online consultation, instead of prescribing. This also suggested that internet healthcare could not entirely replace offline healthcare.

### 4.4 Common health issues addressed via online consultations

Upper respiratory tract infections or fever, gynecological disorders, skin diseases, bone and joint issues, and urological disorders were the top five health issues with the highest number of consultations. Upper respiratory tract infections or fever was mainly caused by mild diseases such as the common cold and rhinitis, which generally lacked urgency and severity. These conditions could typically be adequately described through online consultations with text and images (27). Similar situations exist for skin diseases, users will be able to duplicate offline clinical scenarios to the greatest extent by uploading their disease-related pictures to complete the disease diagnosis (16). Our results also indicated that online consultations, by triaging mild illnesses, could alleviate the strain on offline medical resources to some extent. Similar conclusions were supported by other studies (16, 28). Online consultations for gynecological and urological disorders could alleviate the embarrassment of offline visits. Additionally, the convenience and timeliness of online consultations prompt users to choose this method more often (16). Most cases of joint problems were attributed to osteoarthritis (OA). OA exhibits a higher prevalence among individuals aged 45 years and above in China (29). A considerable proportion of OA patients may not receive long-term treatment due to poor compliance, resistance to medication, or a lack of disease knowledge. Online consultations facilitated access to high-quality medical resources for patients in remote areas who faced challenges in obtaining quality offline medical services (30).

### 4.5 The role of internet healthcare during COVID-19

From 2019 to 2022, the platform encouraged users to engage in online consultations by providing a large number of free services, with a notable increase in pneumonia-related consultations observed in 2022. This trend was reasonable given the emergence of COVID-19 in China at the end of 2019, followed by the rapid implementation of the “dynamic zero-COVID” epidemic prevention and control strategy, which lasted until the end of 2022 (31–33). Our findings suggest that online consultations provided an alternative healthcare access point during the pandemic, helping to alleviate some pressure on offline medical facilities and reduce the risk of cross-infections in healthcare settings. While telemedicine alone could not fully replace in-person care, its increased utilization highlights its potential as a complementary tool in public health emergencies. Given these findings, policymakers could consider further integrating enterprise-led internet healthcare into national health contingency plans, with measures such as insurance reimbursement policies for online consultations, clear physician participation guidelines, and investment in digital healthcare infrastructure. Strengthening these policies could enhance the adaptability of the healthcare system, ensuring more flexible access to medical services during future public health crises.

### 4.6 Gender and age distributions in online consultation participation

Online consultations for females outnumber those for males, especially among women of reproductive age, particularly between 20 and 39 years old. Research indicated that women of reproductive age commonly seek medical information through websites or pregnancy-related apps (34–37). Online consultation, which is not restricted by time or location, can provide practical solutions to improve the health rights of women during pregnancy and postpartum, benefiting both mothers and children (38, 39). Health information can also be obtained through platforms such as websites and applications designed by healthcare professionals, providing decision support for complex pregnancies (40). Traditional Chinese women, who often shouldered greater responsibilities in caring for the elderly and children, might have had more opportunities to utilize online consultation services to address their own health concerns or those of their family members (41). Moreover, female patients in online consultations are often highly educated, showing a high level of acceptance towards online medical consultations. These factors contribute to women of childbearing age, especially pregnant and postpartum women, seeming to be ideal supporters of online consultation (42). Currently, male participation in online consultations was relatively poor, indicating that online consultation platforms have a great potential for development. Optimizing recommendation systems based on the characteristics of male health conditions could attract more male patients to engage in online consultations. The engagement level of patients aged 60 years and above in online consultations was limited, accounting for only 9.20% of the total number of consultations. This may be attributed to technological barriers, limited digital literacy, and usability challenges of online healthcare platforms for older adults (27). According to the 2020 Seventh National Population Census, the proportion of the population aged 60 years and above in China is close to 20% (43). Given the high prevalence of chronic diseases in this age group (44–46), expanding their participation in internet healthcare presents a significant opportunity to improve long-term disease management and access to medical services. To bridge this gap, targeted policies should prioritize enhancing digital accessibility and usability for elderly patients. Key strategies include simplifying telemedicine interfaces, incorporating accessibility features into mobile applications, and implementing government-supported digital literacy programs to equip older adults with the necessary skills for engaging in online healthcare. These measures could significantly increase elderly participation in telemedicine, improve healthcare equity, and enhance the overall effectiveness of internet healthcare services.

### 4.7 Economic factors and pricing disparities in internet healthcare

Variations in consultation pricing across provinces indicate that economic factors influence internet healthcare utilization. Standardizing insurance reimbursement policies for online consultations could help ensure fairer and more affordable access to telemedicine services across regions. Policymakers should also establish clear pricing guidelines and quality control mechanisms to create a more sustainable and regulated online healthcare ecosystem.

### 4.8 Policy recommendations for the sustainable development of internet healthcare

The “*Guidance on Actively Promoting the Payment of Medical Insurance for ‘Internet Plus’ Medical Services*”, issued in November 2020, marked an important step forward in improving the reimbursement policies for internet healthcare services. This policy aimed to address the imbalance in the distribution of medical resources and meet the public's growing demand for convenient healthcare services. Specifically, while some services and medications are reimbursed, gaps remain in coverage, such as for commonly prescribed medications and comprehensive services like chronic disease management (47). The inconsistent management of insurance payments has led to both underutilization and overutilization of services, which could pose risks to the long-term sustainability of internet healthcare (48). To address these gaps, policymakers should consider expanding reimbursement coverage to include a broader range of medications and services, particularly for chronic disease management, remote follow-ups, and specialist consultations. Ensuring that these services are reimbursed would reduce financial barriers for patients, improve access to essential care, and encourage wider patient participation in internet healthcare. Additionally, incorporating tele-consultations and remote follow-ups into insurance schemes could facilitate continuous care for patients, reducing the burden of out-of-pocket expenses. To promote the long-term sustainability of internet healthcare, it is also essential to establish standardized and fair reimbursement policies across provinces. This would help eliminate regional disparities in affordability, promote more equitable access to high-quality healthcare services, and alleviate the strain on offline healthcare systems. Policymakers should also explore incentive mechanisms for primary care providers to engage in telemedicine, ensuring that primary healthcare facilities are better equipped to deliver online services and reduce unnecessary demand for higher-tier hospitals.

Physician participation in online healthcare also requires further policy support. While most online consultations are conducted by senior doctors from tertiary hospitals, a more balanced distribution of online healthcare services across different medical institutions is needed. Policymakers should explore incentive mechanisms to encourage doctors, particularly from primary care facilities, to engage in telemedicine, such as incorporating online consultation hours into professional accreditation and hospital performance evaluations. By strengthening telemedicine-specific training programs, primary care providers could gain more confidence and competence in offering online services, improving patient trust and reducing unnecessary demand for high-tier hospitals.

Moreover, efforts should be made to address the digital divide for elderly patients. The high prevalence of chronic diseases among older adults highlights the need for more accessible and user-friendly telemedicine services. Policymakers should consider simplifying telemedicine platforms through intuitive interfaces, voice-assisted navigation, and one-click access to consultations, as well as government-supported digital literacy programs tailored to elderly users. Community-based initiatives, such as training sessions in local healthcare centers and collaborations with social service organizations, could further enhance adoption among older adults, ensuring that they are not excluded from the benefits of internet healthcare.

The role of enterprise-led internet healthcare in public health emergency response should also be institutionalized. The COVID-19 pandemic demonstrated the critical function of telemedicine in reducing hospital congestion and mitigating cross-infection risks, yet its integration into national contingency plans remains underdeveloped. Moving forward, a structured national telemedicine emergency framework should be established, outlining protocols for rapid mobilization of online healthcare services during crises. This includes standardizing emergency telemedicine service guidelines, expanding investment in digital healthcare infrastructure, and implementing temporary insurance reimbursement mechanisms for telehealth services during public health emergencies.

Addressing these key policy areas would ensure that enterprise-led internet healthcare continues to evolve as a sustainable, scalable, and equitable healthcare model, complementing offline medical services and playing an increasingly vital role in China's healthcare system.

## 5 Limitations

While our study offered valuable insights into the status of enterprise-led internet healthcare in China, there were several limitations. Firstly, we were unable to obtain specific information about patients in online consultations, such as the patients' unique identifiers, and hence were unable to distinguish multiple consultation records from the same patient. Each consultation record was treated as an independent instance, potentially introducing some degree of bias to the results. Future studies could enhance data accuracy by including unique patient identifiers. Moreover, due to the absence of precise patient location information, all geographical statistics were based on the locations of the healthcare institutions to which the online consultation providers belonged. This might not have accurately reflected the redistribution of medical resources geographically by internet healthcare. Future research should incorporate precise patient location data to improve the accuracy of geographic analyses. Additionally, our study only described and analyzed a single enterprise-led internet healthcare platform (Good Doctor Online), which might not have comprehensively reflected the current status of online consultations of internet healthcare in China. Including multiple platforms in future research could provide a broader view of the internet healthcare ecosystem. Lastly, our analysis was limited to the quantity of online consultations, and we did not assess the quality of services provided. Future studies should explore quality measures such as treatment effectiveness, patient satisfaction, and the overall impact of online healthcare.

## 6 Conclusions

Our study profiled the temporal changes and current status of enterprise-led internet healthcare in mainland China using data from Good Doctor Online spanning 15 years (2008–2022). We found that online consultations were predominantly conducted by senior and intermediate-level doctors from tertiary hospitals, with a strong concentration in economically developed regions. The most frequently consulted health issues were upper respiratory tract infections, gynecological disorders, and skin diseases, reflecting the suitability of internet healthcare for non-urgent conditions. Female consultations outnumbered male consultations, and the majority of patients were children under five and young adults aged 20–39.

Our findings highlight the complementary role of enterprise-led internet healthcare in alleviating offline medical resource strain, enhancing access to high-quality healthcare, and mitigating geographical disparities. This was particularly evident during the COVID-19 pandemic, where online consultations helped reduce hospital congestion and cross-infections. While internet healthcare cannot fully replace offline medical services, it serves as a scalable and accessible model that can expand healthcare reach, particularly in underserved regions. To further enhance its impact, we recommend improving accessibility for elderly patients, encouraging participation from senior doctors, optimizing services for male users, expanding access to underserved areas, and broadening insurance reimbursement coverage. Addressing these challenges will help enterprise-led internet healthcare evolve as an effective solution for improving healthcare delivery and reducing disparities.

## Data Availability

The original contributions presented in the study are included in the article/Supplementary Material, further inquiries can be directed to the corresponding author.

## References

[1] Eysenbach G. What is e-health? J Med Internet Res. (2001) 3(2):e833. 10.2196/jmir.3.2.e20 PMC1761894 11720962

[2] Sood S Mbarika V Jugoo S Dookhy R Doarn CR Prakash N What is telemedicine? A collection of 104 peer-reviewed perspectives and theoretical underpinnings. Telemed e-Health. (2007) 13(5):573–90. 10.1089/tmj.2006.0073 17999619

[3] SCotPsRo C. Guidance on Actively Promoting the Action of “Internet plus”. 2015 1 October 2022. Available online at: http://www.gov.cn/zhengce/content/2015-07/04/content_10002.htm (Accessed October 01, 2022).

[4] NHCaNAoTCMotPsRo C. Measures for the Administration of Internet Diagnosis and Treatment (for Trial Implementation). (2018).

[5] NHS A. Guidance on Actively Promoting the Payment of Medical Insurance for “Internet Plus” Medical Services. 2020 1 October 2022.

[6] Han Y Lie RK Guo R. The internet hospital as a telehealth model in China: systematic search and content analysis. J Med Internet Res. (2020) 22(7):e17995. 10.2196/17995 32723721 PMC7424477

[7] Zhang RL Wang G. “Internet +” medical service supply: model comparison and optimal pathway (in Chinese). Health Economics Research. (2022) 39(3):32–7. 10.14055/j.cnki.33-1056/f.2022.03.008

[8] Anand S Fan VY Zhang J Zhang L Ke Y Dong Z China’s human resources for health: quantity, quality, and distribution. Lancet. (2008) 372(9651):1774–81. 10.1016/S0140-6736(08)61363-X 18930528

[9] Sun J Luo H. Evaluation on equality and efficiency of health resources allocation and health services utilization in China. Int J Equity Health. (2017) 16(1):1–8. 10.1186/s12939-016-0499-1 28709422 PMC5513103

[10] Li D Zhou Z Si Y Xu Y Shen C Wang Y Unequal distribution of health human resource in mainland China: what are the determinants from a comprehensive perspective? Int J Equity Health. (2018) 17(1):1–12. 10.1186/s12939-017-0710-z 29486791 PMC5830142

[11] Chai K-C Zhang Y-B Chang K-C. Regional disparity of medical resources and its effect on mortality rates in China. Front Public Health. (2020) 8:8. 10.3389/fpubh.2020.00008 32117848 PMC7011092

[12] Gao J Fan C Chen B Fan Z Li L Wang L Telemedicine is becoming an increasingly popular way to resolve the unequal distribution of healthcare resources: evidence from China. Front Public Health. (2022) 10:916303. 10.3389/fpubh.2022.916303 35874991 PMC9301261

[13] Gong K Xu Z Cai Z Chen Y Wang Z. Internet hospitals help prevent and control the epidemic of COVID-19 in China: multicenter user profiling study. J Med Internet Res. (2020) 22(4):e18908. 10.2196/18908 32250962 PMC7159055

[14] Hong Z Li N Li D Li J Li B Xiong W Telemedicine during the COVID-19 pandemic: experiences from western China. J Med Internet Res. (2020) 22(5):e19577. 10.2196/19577 32349962 PMC7212818

[15] Perisetti A Goyal H. Successful distancing: telemedicine in gastroenterology and hepatology during the COVID-19 pandemic. Dig Dis Sci. (2021) 66:945–53. 10.1007/s10620-021-06874-x 33655456 PMC7925138

[16] Jiang X Xie H Tang R Du Y Li T Gao J Characteristics of online health care services from China’s largest online medical platform: cross-sectional survey study. J Med Internet Res. (2021) 23(4):e25817. 10.2196/25817 33729985 PMC8051434

[17] Xu X Cai Y Wu S Guo J Yang L Lan J Assessment of internet hospitals in China during the COVID-19 pandemic: national cross-sectional data analysis study. J Med Internet Res. (2021) 23(1):e21825. 10.2196/21825 33417586 PMC7819672

[18] Tu J Wang C Wu S. The internet hospital: an emerging innovation in China. Lancet Glob Health. (2015) 3(8):e445–6. eng. 10.1016/s2214-109x(15)00042-x 26187488 PMC7129805

[19] Wang X Wang J Du X Yu L Zhou Y Zhou S Telemedicine during the COVID-19 epidemic improves outcomes in children with tuberous sclerosis complex: a 1206 visits retrospective cohort study. CNS Neurosci Ther. (2024) 30(6):e14549. 10.1111/cns.14549 38031962 PMC11163188

[20] Sang L Song L. The current status of the use of internet hospitals for outpatients with pain: retrospective study. J Med Internet Res. (2023) 25:e44759. 10.2196/44759 37695652 PMC10520772

[21] Cui F He X Zhai Y Lyu M Shi J Sun D Application of telemedicine services based on a regional telemedicine platform in China from 2014 to 2020: longitudinal trend analysis. J Med Internet Res. (2021) 23(7):e28009. 10.2196/28009 34255686 PMC8314158

[22] Du Y Zhou Q Cheng W Zhang Z Hoelzer S Liang Y Factors influencing adoption and use of telemedicine services in rural areas of China: mixed methods study. JMIR Public Health and Surveillance. (2022) 8(12):e40771. 10.2196/40771 36563026 PMC9823570

[23] Li L Liu G Xu W Zhang Y He M. Effects of internet hospital consultations on psychological burdens and disease knowledge during the early outbreak of COVID-19 in China: cross-sectional survey study. J Med Internet Res. (2020) 22(8):e19551. 10.2196/19551 32687061 PMC7427983

[24] Online GD. Introduction of Good Doctor Online. 2024 February 7, 2024. Available online at: https://www.haodf.com/info/aboutus.php (Accessed February 07, 2024).

[25] Yao H Zhan C Sha X. Current situation and distribution equality of public health resource in China. Arch Public Health. (2020) 78(1):1–7. 10.1186/s13690-019-0383-8 32983449 PMC7507592

[26] Wang Y Liu Y Shi Y Yu Y Yang J. User perceptions of virtual hospital apps in China: systematic search. JMIR Mhealth Uhealth. (2020) 8(8):e19487. 10.2196/19487 32687480 PMC7450379

[27] Wang X Huang T. Study on characteristics of patients and agent consultation in online healthcare consultation: a case study of dxy. com. Chinese Journal of Health Policy. (2021) 14(9):8. 10.3969/j.issn.1674-2982.2021.09.009

[28] Katayama Y Kiyohara K Hirose T Matsuyama T Ishida K Nakao S A mobile app for self-triage for pediatric emergency patients in Japan: 4 year descriptive epidemiological study. JMIR Pediatrics and Parenting. (2021) 4(2):e27581. 10.2196/27581 34255709 PMC8280828

[29] Tang X Wang S Zhan S Niu J Tao K Zhang Y The prevalence of symptomatic knee osteoarthritis in China: results from the China health and retirement longitudinal study. Arthritis Rheumatol. (2016) 68(3):648–53. 10.1002/art.39465 26474054

[30] Huang Z Pan X Deng W Huang Z Huang Y Huang X Implementation of telemedicine for knee osteoarthritis: study protocol for a randomized controlled trial. Trials. (2018) 19(1):1–8. 10.1186/s13063-018-2625-4 29665830 PMC5904993

[31] Li Q Guan X Wu P Wang X Zhou L Tong Y Early transmission dynamics in Wuhan, China, of novel coronavirus–infected pneumonia. N Engl J Med. (2020) 382(13):1199–207. 10.1056/NEJMoa2001316 31995857 PMC7121484

[32] Ge J. The COVID-19 pandemic in China: from dynamic zero-COVID to current policy. Herz. (2023) 48(3):226–8. 10.1007/s00059-023-05183-5 37294456 PMC10252163

[33] National Health Commission of the People's Republic of China. Notice on Further Optimizing the Implementation of COVID-19 Prevention and Control Measures. 2022 February 7, 2024. Available online at: http://www.nhc.gov.cn/xcs/gzzcwj/202212/8278e7a7aee34e5bb378f0e0fc94e0f0.shtml (Accessed February 07, 2024).

[34] Wallwiener S Müller M Doster A Laserer W Reck C Pauluschke-Fröhlich J Pregnancy eHealth and mHealth: user proportions and characteristics of pregnant women using web-based information sources—a cross-sectional study. Arch Gynecol Obstet. (2016) 294:937–44. 10.1007/s00404-016-4093-y 27084763

[35] Bert F Gualano MR Brusaferro S De Vito E De Waure C La Torre G Pregnancy e-health: a multicenter Italian cross-sectional study on internet use and decision-making among pregnant women. J Epidemiol Community Health. (2013) 67(12):1013–8. 10.1136/jech-2013-202584 24072743

[36] Sayakhot P Carolan-Olah M. Internet use by pregnant women seeking pregnancy-related information: a systematic review. BMC Pregnancy Childbirth. (2016) 16(1):1–10. 10.1186/s12884-015-0735-5 27021727 PMC4810511

[37] Scaioli G Bert F Galis V Brusaferro S De Vito E La Torre G Pregnancy and internet: sociodemographic and geographic differences in e-health practice. Results from an Italian multicenter study. Public Health. (2015) 129(9):1258–66. 10.1016/j.puhe.2015.06.012 26210071

[38] Sherifali D Nerenberg KA Wilson S Semeniuk K Ali MU Redman LM The effectiveness of eHealth technologies on weight management in pregnant and postpartum women: systematic review and meta-analysis. J Med Internet Res. (2017) 19(10):e337. 10.2196/jmir.8006 29030327 PMC5660296

[39] Lettieri E Fumagalli LP Radaelli G Bertele’ P Vogt J Hammerschmidt R Empowering patients through eHealth: a case report of a pan-European project. BMC Health Serv Res. (2015) 15:1–12. 10.1186/s12913-015-0983-0 26242863 PMC4526304

[40] Vlemmix F Warendorf JK Rosman AN Kok M Mol BWJ Morris JM Decision aids to improve informed decision-making in pregnancy care: a systematic review. BJOG. (2013) 120(3):257–66. 10.1111/1471-0528.12060 23145991

[41] Ge F Qian H Lei J Ni Y Li Q Wang S Experiences and challenges of emerging online health services combating COVID-19 in China: retrospective, cross-sectional study of internet hospitals. JMIR Med Inform. (2022) 10(6):e37042. 10.2196/37042 35500013 PMC9162135

[42] Van Den Heuvel JF Groenhof TK Veerbeek JH Van Solinge WW Lely AT Franx A Ehealth as the next-generation perinatal care: an overview of the literature. J Med Internet Res. (2018) 20(6):e202. 10.2196/jmir.9262 29871855 PMC6008510

[43] Census OotLGotSCftSNP. China Population Census Yearbook 2020. 2020 February 7, 2024. Available online at: https://www.stats.gov.cn/sj/pcsj/rkpc/7rp/zk/indexch.htm (Accessed February 07, 2024).

[44] Wu F Narimatsu H Li X Nakamura S Sho R Zhao G Non-communicable diseases control in China and Japan. Global Health. (2017) 13(1):91. 10.1186/s12992-017-0315-8 29262849 PMC5738724

[45] Monaco A Maggi S De Cola P Hassan TA Palmer K Donde S. Information and communication technology for increasing healthy ageing in people with non-communicable diseases: identifying challenges and further areas for development. Aging Clin Exp Res. (2019) 31(11):1689–93. 10.1007/s40520-019-01258-8 31317518 PMC6825021

[46] Zhou M Wang H Zhu J Chen W Wang L Liu S Cause-specific mortality for 240 causes in China during 1990–2013: a systematic subnational analysis for the global burden of disease study 2013. Lancet. (2016) 387(10015):251–72. 10.1016/S0140-6736(15)00551-6 26510778

[47] Lin XY Li J Ti SM Qi JY Yang HL. Problems and countermeasures in medical insurance payment for “internet+” medical services (in Chinese). Chin J Med Manag Sci. (2021) 11(2):35–7. 10.3969/jssn.2095-7432.2021.02.006

[48] Cui WB Gu ST Cun DL Zhang S Yu GJ. Risks and management suggestions after “internet +” medical services are included in medical insurance payments (in Chinese). Chin Hospital. (2020) 24(3):10–2. 10.19660/j.issn.1671-0592.2020.03.04

