# Supplementary material for: Enterprise-led internet healthcare provision in China: insights from a leading platform
Source: Front Digit Health. 2025 Mar 3;7:1491183. doi: 10.3389/fdgth.2025.1491183 (PMC11911379; doi:10.3389/fdgth.2025.1491183)
Supplement: Supplementary file 1 [file Datasheet1.docx]

APPENDIX

TABLE OF CONTENTS

Figure S1: Data acquisition process diagram 2

Appendix 1: The list of acquired variable information from the internet hospital platform 3

Figure S2: The number of doctors providing online consultations 4

Figure S3: The number of consultations provided per doctor 6

Figure S4: The proportion of free consultations grouped by year and department 8

Table S1: Characteristics of the patients seeking online consultations 9

Table S2: The characteristics of doctors providing online consultations in each province and their affiliated healthcare institutions 10

Table S3: The characteristics of each department providing online consultations 12

Table S4: The number and proportions of doctors and consultations categorized by healthcare institutions and professional titles 14

Table S5: The number and proportions of online consultations provided from 2008 to 2022 15

Table S6: The number of consultations provided per active doctor from 2008 to 2022 19

Table S7: The consultation price from 2008 to 2022 24

### Figure S1: Data acquisition process diagram


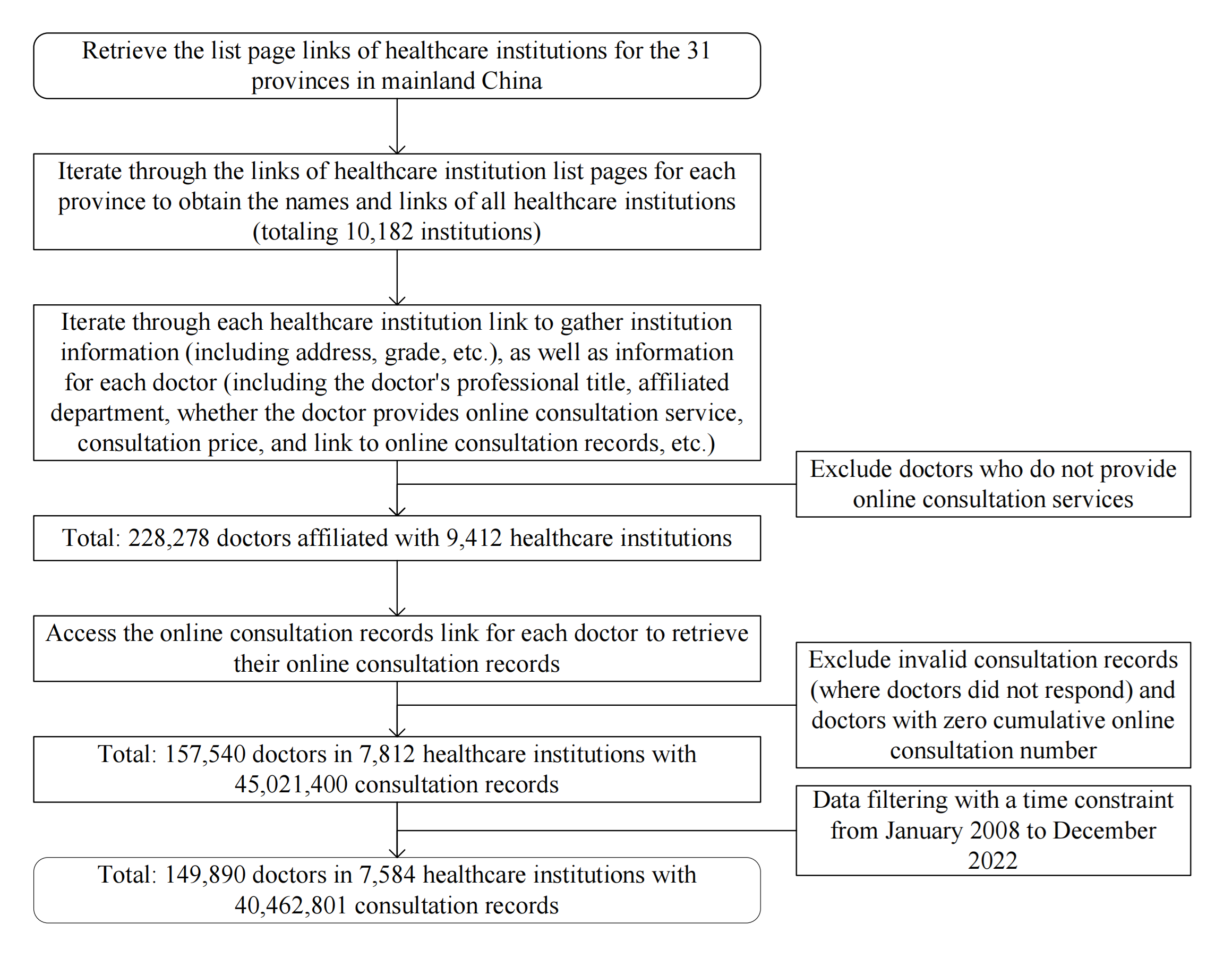


### Appendix 1: The list of acquired variable information from the internet hospital platform

**List of acquired variable information**

| **Category** | **Details** |
| --- | --- |
| Doctor | Doctor ID |
|  | Professional title |
|  | Department |
|  | Name of healthcare institution |
| Healthcare institution | Name of healthcare institution |
|  | Address of healthcare institution |
|  | Classification or grade of healthcare institution |
| Consultation record | Doctor ID |
|  | Patient gender |
|  | Patient age |
|  | Consultation department |
|  | Consultation health issue |
|  | Consultation date |
|  | Consultation price |

### Figure S2: The number of doctors providing online consultations


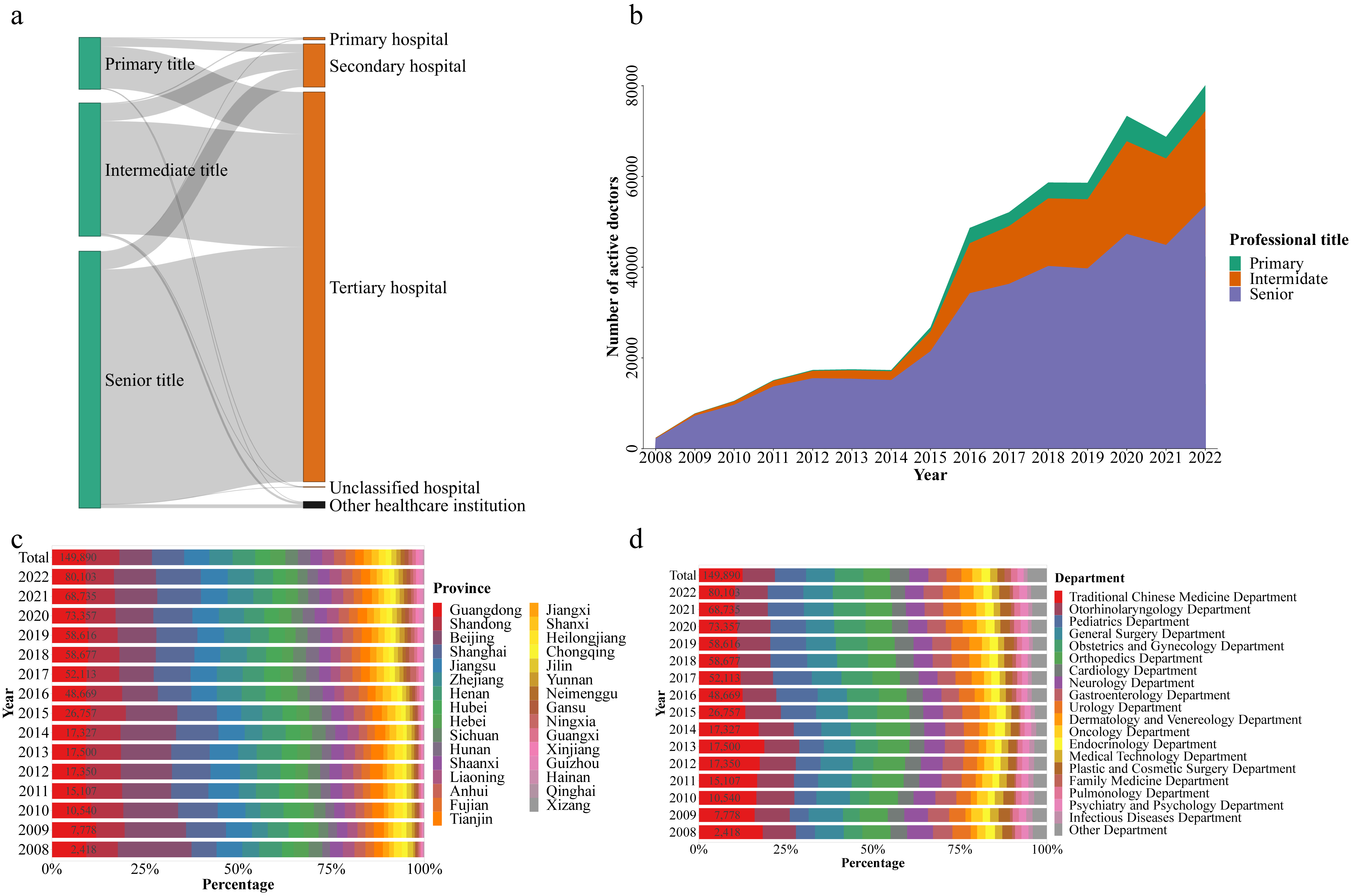


The composition of doctors, grouped according to their professional titles and the healthcare institutions they were employed by (a). The number of active doctors providing online consultations by professional title (b), province (c), and department (d), for each year. An active doctor refers to a physician who has provided at least one online consultation service within a specified period, such as one year.

### Figure S3: The number of consultations provided per doctor


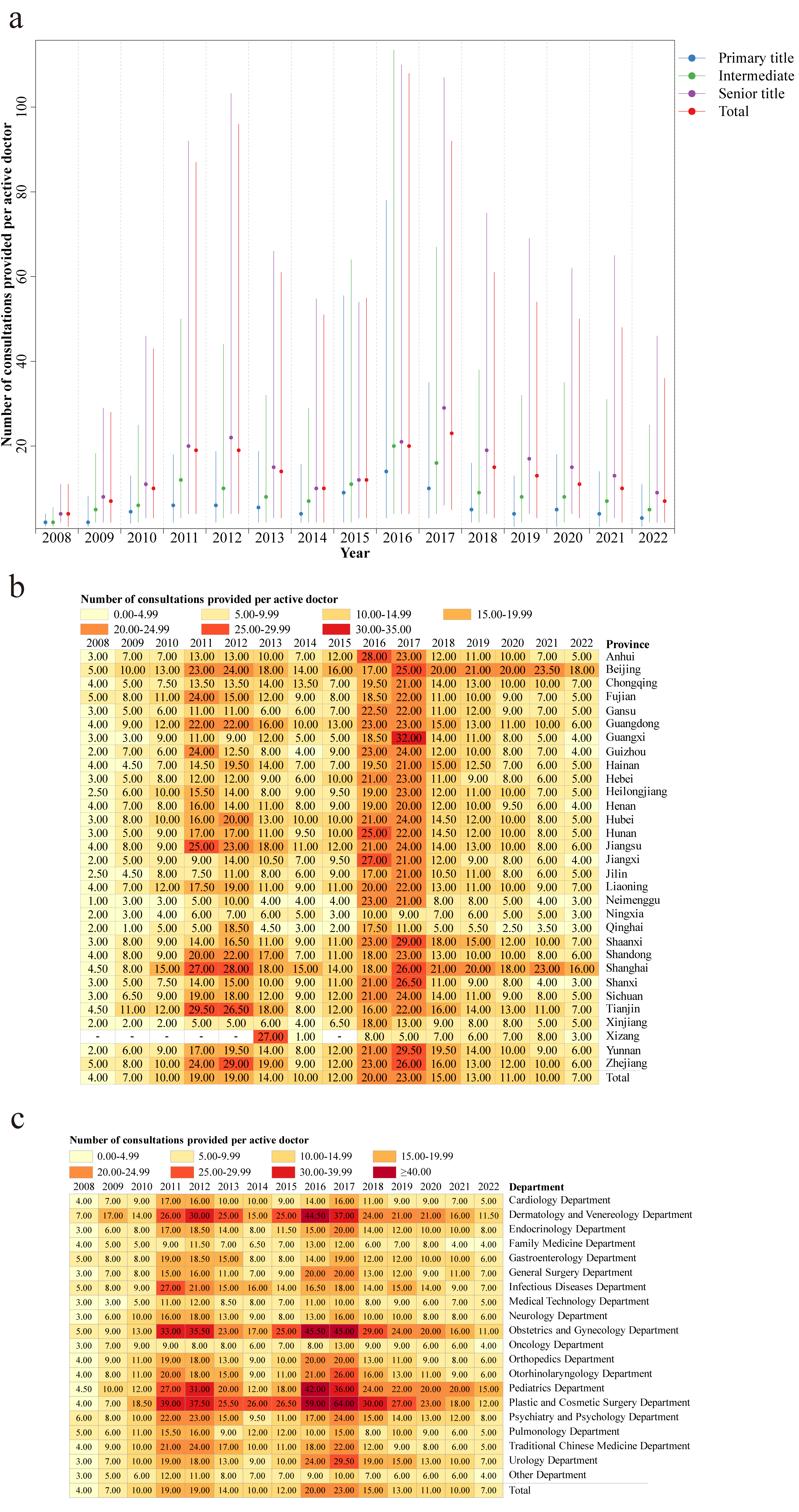


The number of consultations provided per doctor by professional title (a), province (b), and department (c), for each year.

### Figure S4: The proportion of free consultations grouped by year and department


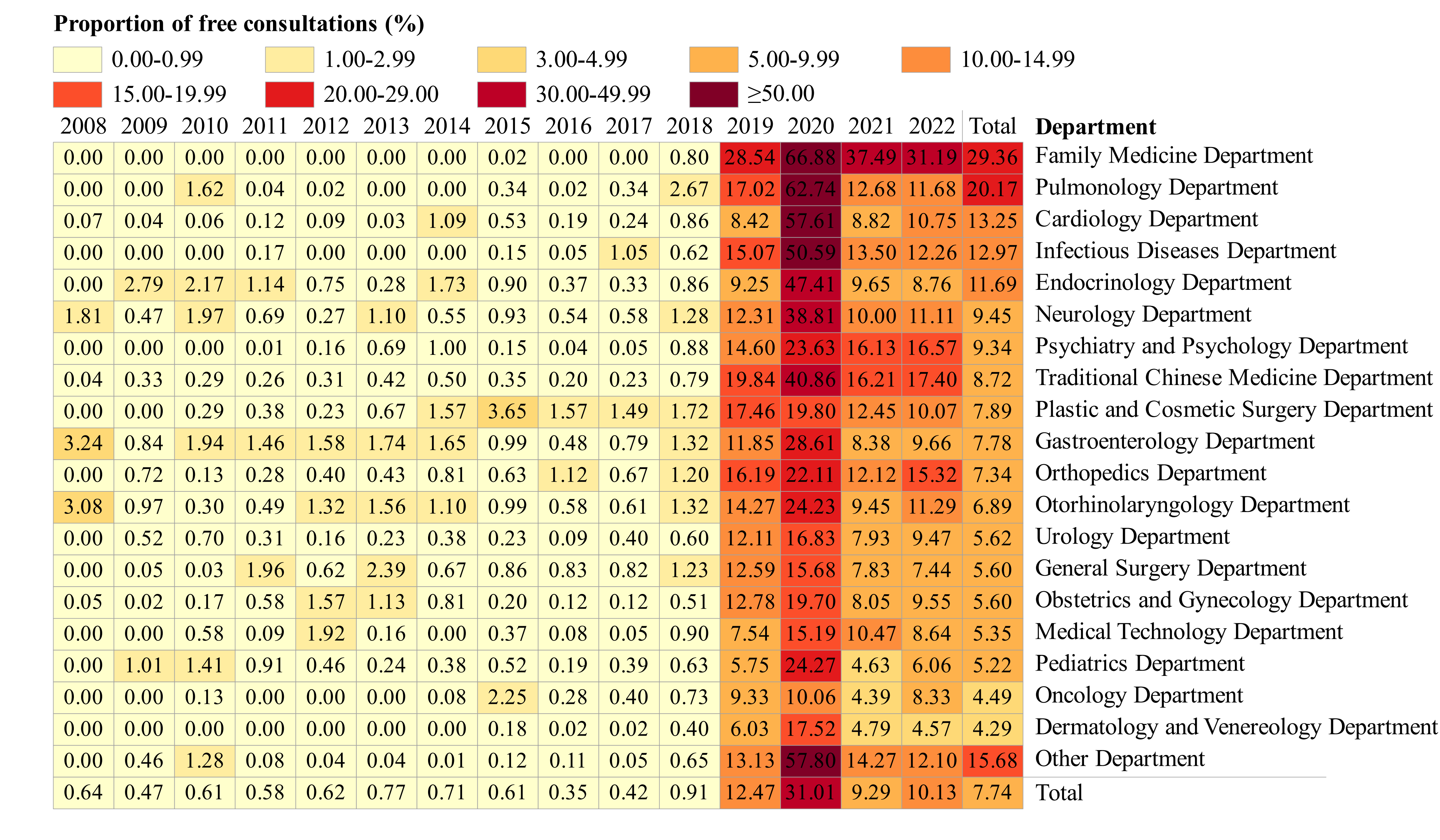


### Table S1: Characteristics of the patients seeking online consultations

| **Year** | **Age of patients, *median* (*IQR*) years** | | |  | **Number of consultations, *n*** | | |  | **Male-to-Female consultation ratio** |
| --- | --- | --- | --- | --- | --- | --- | --- | --- | --- |
|  | **Female** | **Male** | **Total** |  | Female | Male | Total |  |  |
| 2008 | 30.00 (22.00, 45.00) | 28.00 (17.00, 45.00) | 29.00 (21.00, 45.00) |  | 14289 | 13204 | 27493 |  | 0.92 |
| 2009 | 30.00 (23.00, 47.00) | 30.00 (20.00, 48.00) | 30.00 (22.00, 47.00) |  | 137212 | 121348 | 258560 |  | 0.88 |
| 2010 | 31.00 (23.00, 47.00) | 30.00 (20.00, 49.00) | 31.00 (22.00, 48.00) |  | 283053 | 248849 | 531902 |  | 0.88 |
| 2011 | 30.00 (22.00, 46.00) | 29.00 (17.00, 48.00) | 29.00 (21.00, 47.00) |  | 650550 | 561020 | 1211570 |  | 0.86 |
| 2012 | 29.00 (22.00, 45.00) | 28.00 (17.00, 47.00) | 29.00 (21.00, 46.00) |  | 812676 | 705579 | 1518255 |  | 0.87 |
| 2013 | 28.00 (22.00, 42.00) | 27.00 (18.00, 45.00) | 28.00 (21.00, 43.00) |  | 566220 | 481138 | 1047358 |  | 0.85 |
| 2014 | 28.00 (22.00, 40.00) | 27.00 (17.00, 43.00) | 28.00 (21.00, 41.00) |  | 531646 | 395887 | 927533 |  | 0.74 |
| 2015 | 28.00 (22.00, 38.00) | 26.00 (11.00, 40.00) | 27.00 (20.00, 38.00) |  | 1120179 | 821319 | 1941498 |  | 0.73 |
| 2016 | 27.00 (21.00, 36.00) | 25.00 (6.00, 38.00) | 27.00 (17.00, 37.00) |  | 3653410 | 2619493 | 6272903 |  | 0.72 |
| 2017 | 29.00 (21.00, 40.00) | 27.00 (7.08, 42.00) | 28.00 (17.00, 41.00) |  | 2627203 | 1958423 | 4585626 |  | 0.75 |
| 2018 | 30.00 (23.00, 43.00) | 29.00 (9.17, 47.00) | 30.00 (19.00, 45.00) |  | 2074964 | 1576951 | 3651915 |  | 0.76 |
| 2019 | 31.00 (23.00, 44.00) | 29.00 (10.00, 47.00) | 30.00 (20.00, 45.00) |  | 1879676 | 1393467 | 3273143 |  | 0.74 |
| 2020 | 30.00 (22.00, 41.00) | 29.00 (13.00, 44.00) | 30.00 (20.00, 42.00) |  | 3020804 | 2240140 | 5260944 |  | 0.74 |
| 2021 | 32.00 (24.00, 45.00) | 30.00 (10.00, 49.00) | 31.00 (20.00, 47.00) |  | 2660694 | 1966479 | 4627173 |  | 0.74 |
| 2022 | 32.00 (24.00, 46.00) | 31.00 (9.83, 50.00) | 32.00 (19.00, 48.00) |  | 3066852 | 2260076 | 5326928 |  | 0.74 |
| Total | 30.00 (22.00, 42.00) | 28.00 (10.00, 45.00) | 29.00 (19.00, 43.00) |  | 23099428 | 17363373 | 40462801 |  | 0.75 |

### Table S2: The characteristics of doctors providing online consultations in each province and their affiliated healthcare institutions

| **Province** | **Number of doctors providing online consultations, *n* (%)** | | | | **Number of consultations, *n* (%)** | **Number of consultations provided per doctor, *median* (*IQR*)** | **Consultation price, *median* (*IQR*) RMB** | **Healthcare institution** | | | | | |
| --- | --- | --- | --- | --- | --- | --- | --- | --- | --- | --- | --- | --- | --- |
|  | **Primary title** | **Intermediate title** | **Senior title** | **Total** |  |  |  | **Hospital** | | | | **Other healthcare institution** | **Total** |
|  |  |  |  |  |  |  |  | **Primary hospital** | **Secondary hospital** | **Tertiary hospital** | **Unclassified hospital** |  |  |
| Anhui | 763 (16.18) | 1379 (29.23) | 2575 (54.59) | 4717(3.15) | 1199784 (2.97) | 26.00 (5.00, 186.00) | 29.00 (9.00, 50.00) | 23 (8.61) | 147 (55.06) | 85 (31.84) | 1 (0.37) | 11 (4.12) | 267 (3.52) |
| Beijing | 1038 (7.94) | 3560 (27.24) | 8471 (64.82) | 13069(8.72) | 5377044 (13.29) | 77.00 (10.00, 469.00) | 100.00 (50.00, 200.00) | 44 (15.17) | 68 (23.45) | 109 (37.59) | 2 (0.69) | 67 (23.10) | 290 (3.82) |
| Chongqing | 332 (14.66) | 826 (36.47) | 1107 (48.87) | 2265(1.51) | 561725 (1.39) | 21.00 (4.00, 158.00) | 37.50 (11.50, 80.00) | 6 (4.11) | 83 (56.85) | 53 (36.30) | 0 (0.00) | 4 (2.74) | 146 (1.93) |
| Fujian | 516 (13.75) | 1188 (31.65) | 2049 (54.60) | 3753(2.50) | 821536 (2.03) | 20.00 (4.00, 138.00) | 33.00 (10.00, 66.00) | 14 (6.45) | 104 (47.93) | 83 (38.25) | 2 (0.92) | 14 (6.45) | 217 (2.86) |
| Gansu | 236 (15.08) | 470 (30.03) | 859 (54.89) | 1565(1.04) | 299006 (0.74) | 19.00 (3.00, 128.00) | 29.00 (9.00, 45.00) | 9 (5.49) | 103 (62.80) | 47 (28.66) | 0 (0.00) | 5 (3.05) | 164 (2.16) |
| Guangdong | 1786 (13.05) | 3891 (28.43) | 8007 (58.51) | 13684(9.13) | 3854956 (9.53) | 30.00 (5.00, 213.00) | 50.00 (26.00, 100.00) | 34 (5.98) | 262 (46.05) | 239 (42.00) | 4 (0.70) | 30 (5.27) | 569 (7.50) |
| Guangxi | 166 (11.67) | 431 (30.31) | 825 (58.02) | 1422(0.95) | 236372 (0.58) | 15.00 (3.00, 87.00) | 29.00 (9.00, 49.00) | 4 (2.48) | 74 (45.96) | 79 (49.07) | 0 (0.00) | 4 (2.48) | 161 (2.12) |
| Guizhou | 155 (11.99) | 439 (33.95) | 699 (54.06) | 1293(0.86) | 209300 (0.52) | 18.00 (4.00, 95.00) | 29.00 (9.00, 50.00) | 2 (1.56) | 65 (50.78) | 57 (44.53) | 0 (0.00) | 4 (3.13) | 128 (1.69) |
| Hainan | 66 (12.62) | 131 (25.05) | 326 (62.33) | 523(0.35) | 114331 (0.28) | 13.00 (3.00, 96.50) | 30.00 (9.00, 50.00) | 0 (0.00) | 17 (37.78) | 24 (53.33) | 0 (0.00) | 4 (8.89) | 45 (0.59) |
| Hebei | 878 (14.46) | 2113 (34.80) | 3081 (50.74) | 6072(4.05) | 1394936 (3.45) | 20.00 (4.00, 141.00) | 18.00 (9.00, 35.00) | 23 (5.39) | 303 (70.96) | 86 (20.14) | 0 (0.00) | 15 (3.51) | 427 (5.63) |
| Heilongjiang | 265 (9.49) | 659 (23.59) | 1869 (66.92) | 2793(1.86) | 617497 (1.53) | 19.00 (4.00, 149.00) | 30.00 (9.00, 60.00) | 8 (3.98) | 91 (45.27) | 97 (48.26) | 0 (0.00) | 5 (2.49) | 201 (2.65) |
| Henan | 1260 (13.93) | 3197 (35.36) | 4585 (50.71) | 9042(6.03) | 2036447 (5.03) | 21.00 (4.00, 144.00) | 25.00 (9.00, 50.00) | 58 (12.16) | 281 (58.91) | 130 (27.25) | 0 (0.00) | 8 (1.68) | 477 (6.29) |
| Hubei | 650 (10.68) | 1926 (31.64) | 3511 (57.68) | 6087(4.06) | 1532231 (3.79) | 25.00 (5.00, 173.00) | 37.50 (15.00, 80.00) | 19 (6.38) | 144 (48.32) | 125 (41.95) | 0 (0.00) | 10 (3.36) | 298 (3.93) |
| Hunan | 690 (13.86) | 1401 (28.14) | 2887 (58.00) | 4978(3.32) | 1178497 (2.91) | 27.00 (4.00, 168.75) | 48.00 (15.00, 99.00) | 20 (5.88) | 191 (56.18) | 118 (34.71) | 1 (0.29) | 10 (2.94) | 340 (4.48) |
| Jiangsu | 909 (8.97) | 2247 (22.17) | 6978 (68.86) | 10134(6.76) | 2756654 (6.81) | 30.00 (5.00, 202.00) | 40.00 (15.00, 94.50) | 21 (5.29) | 146 (36.78) | 198 (49.87) | 0 (0.00) | 32 (8.06) | 397 (5.23) |
| Jiangxi | 609 (19.21) | 1152 (36.33) | 1410 (44.47) | 3171(2.12) | 605442 (1.50) | 17.00 (3.00, 119.50) | 29.00 (9.00, 50.00) | 13 (4.78) | 159 (58.46) | 96 (35.29) | 0 (0.00) | 4 (1.47) | 272 (3.59) |
| Jilin | 195 (9.60) | 611 (30.07) | 1226 (60.33) | 2032(1.36) | 352763 (0.87) | 15.00 (4.00, 94.00) | 30.00 (9.00, 60.00) | 7 (4.93) | 75 (52.82) | 54 (38.03) | 0 (0.00) | 6 (4.23) | 142 (1.87) |
| Liaoning | 392 (8.26) | 1193 (25.15) | 3159 (66.59) | 4744(3.16) | 1134799 (2.80) | 25.00 (4.00, 169.00) | 30.00 (9.00, 69.00) | 10 (3.79) | 97 (36.74) | 147 (55.68) | 0 (0.00) | 10 (3.79) | 264 (3.48) |
| Neimenggu | 164 (10.20) | 387 (24.07) | 1057 (65.73) | 1608(1.07) | 263568 (0.65) | 10.00 (3.00, 61.00) | 19.00 (9.00, 37.50) | 7 (4.67) | 65 (43.33) | 70 (46.67) | 0 (0.00) | 8 (5.33) | 150 (1.98) |
| Ningxia | 208 (13.68) | 421 (27.68) | 892 (58.65) | 1521(1.01) | 165041 (0.41) | 10.00 (2.00, 61.00) | 29.00 (10.00, 37.50) | 6 (8.82) | 33 (48.53) | 22 (32.35) | 0 (0.00) | 7 (10.29) | 68 (0.90) |
| Qinghai | 24 (10.76) | 61 (27.35) | 138 (61.88) | 223(0.15) | 11067 (0.03) | 7.00 (2.00, 30.50) | 10.00 (9.00, 29.00) | 0 (0.00) | 6 (27.27) | 16 (72.73) | 0 (0.00) | 0 (0.00) | 22 (0.29) |
| Shaanxi | 623 (13.03) | 1563 (32.69) | 2595 (54.28) | 4781(3.19) | 1344123 (3.32) | 31.00 (5.00, 232.00) | 39.00 (12.50, 90.00) | 10 (3.89) | 163 (63.42) | 77 (29.96) | 0 (0.00) | 7 (2.72) | 257 (3.39) |
| Shandong | 1790 (13.25) | 4827 (35.73) | 6891 (51.01) | 13508(9.01) | 3428676 (8.47) | 24.00 (4.00, 172.00) | 29.00 (9.00, 50.00) | 101 (15.56) | 303 (46.69) | 210 (32.36) | 1 (0.15) | 34 (5.24) | 649 (8.56) |
| Shanghai | 952 (7.35) | 4032 (31.12) | 7974 (61.54) | 12958(8.65) | 4968948 (12.28) | 72.00 (10.00, 427.00) | 99.00 (49.00, 199.00) | 28 (12.44) | 94 (41.78) | 64 (28.44) | 14 (6.22) | 25 (11.11) | 225 (2.97) |
| Shanxi | 350 (12.12) | 828 (28.67) | 1710 (59.21) | 2888(1.93) | 672299 (1.66) | 20.00 (4.00, 153.00) | 25.00 (9.00, 50.00) | 9 (3.98) | 142 (62.83) | 67 (29.65) | 1 (0.44) | 7 (3.10) | 226 (2.98) |
| Sichuan | 712 (14.07) | 1733 (34.24) | 2616 (51.69) | 5061(3.38) | 1241780 (3.07) | 24.00 (4.00, 161.00) | 37.50 (12.50, 90.00) | 16 (4.11) | 126 (32.39) | 234 (60.15) | 1 (0.26) | 12 (3.08) | 389 (5.13) |
| Tianjin | 342 (9.81) | 1061 (30.44) | 2083 (59.75) | 3486(2.33) | 991971 (2.45) | 38.00 (6.00, 257.75) | 50.00 (25.00, 100.00) | 15 (14.85) | 35 (34.65) | 47 (46.53) | 0 (0.00) | 4 (3.96) | 101 (1.33) |
| Xinjiang | 218 (15.38) | 463 (32.67) | 736 (51.94) | 1417(0.95) | 162832 (0.40) | 12.00 (3.00, 54.00) | 20.00 (9.00, 50.00) | 7 (4.73) | 86 (58.11) | 52 (35.14) | 0 (0.00) | 3 (2.03) | 148 (1.95) |
| Xizang | 13 (26.53) | 19 (38.78) | 17 (34.69) | 49(0.03) | 5754 (0.01) | 8.00 (3.00, 31.00) | 20.00 (0.00, 50.00) | 0 (0.00) | 1 (12.50) | 7 (87.50) | 0 (0.00) | 0 (0.00) | 8 (0.11) |
| Yunnan | 299 (17.82) | 517 (30.81) | 862 (51.37) | 1678(1.12) | 352481 (0.87) | 22.00 (4.00, 151.75) | 35.00 (11.50, 69.00) | 1 (0.67) | 69 (46.00) | 76 (50.67) | 0 (0.00) | 4 (2.67) | 150 (1.98) |
| Zhejiang | 972 (10.38) | 2468 (26.35) | 5928 (63.28) | 9368(6.25) | 2570941 (6.35) | 31.00 (6.00, 216.00) | 50.00 (20.00, 100.00) | 11 (2.85) | 175 (45.34) | 162 (41.97) | 6 (1.55) | 32 (8.29) | 386 (5.09) |
| Total | 17573 (11.72) | 45194 (30.15) | 87123 (58.12) | 149890(100.00) | 40462801 (100.00) | 29.00 (5.00, 203.00) | 49.00 (15.00, 100.00) | 526 (6.94) | 3708 (48.89) | 2931 (38.65) | 33 (0.44) | 386 (5.09) | 7584 (100.00) |

### Table S3: The characteristics of each department providing online consultations

| **Department** | **Number of doctors, *n* (*%*)** | **Number of consultations, *n* (*%*)** | **Number of consultations provided per doctor, *median* (*IQR*)** | **Consultation price, *median* (*IQR*) RMB** | **Number of doctors providing online consultations by professional title, *n* (%)** | | |
| --- | --- | --- | --- | --- | --- | --- | --- |
|  |  |  |  |  | **Primary title** | **Intermediate title** | **Senior title** |
| Cardiology Department | 8343(5.57) | 1453551 (3.59) | 19.00 (4.00, 102.00) | 37.50 (10.00, 100.00) | 890 (10.67) | 2335 (27.99) | 5118 (61.34) |
| Dermatology and Venereology Department | 4671(3.12) | 2111999 (5.22) | 78.00 (12.00, 511.00) | 50.00 (25.00, 100.00) | 656 (14.04) | 1645 (35.22) | 2370 (50.74) |
| Endocrinology Department | 3748(2.50) | 953964 (2.36) | 23.00 (5.00, 166.25) | 50.00 (18.00, 120.00) | 311 (8.30) | 1014 (27.05) | 2423 (64.65) |
| Family Medicine Department | 2782(1.86) | 476558 (1.18) | 12.00 (3.00, 72.00) | 9.00 (0.00, 20.00) | 632 (22.72) | 1098 (39.47) | 1052 (37.81) |
| Gastroenterology Department | 7816(5.21) | 1840663 (4.55) | 26.00 (5.00, 174.00) | 50.00 (20.00, 100.00) | 668 (8.55) | 1997 (25.55) | 5151 (65.90) |
| General Surgery Department | 12360(8.25) | 3043463 (7.52) | 28.00 (4.00, 195.00) | 50.00 (19.00, 100.00) | 1237 (10.01) | 3605 (29.17) | 7518 (60.83) |
| Infectious Diseases Department | 1695(1.13) | 495564 (1.22) | 32.00 (5.00, 193.00) | 50.00 (10.00, 100.00) | 149 (8.79) | 405 (23.89) | 1141 (67.32) |
| Medical Technology Department | 3116(2.08) | 575082 (1.42) | 10.00 (2.00, 72.25) | 50.00 (20.00, 100.00) | 333 (10.69) | 891 (28.59) | 1892 (60.72) |
| Neurology Department | 8280(5.52) | 1722058 (4.26) | 19.00 (4.00, 136.00) | 50.00 (19.00, 125.00) | 731 (8.83) | 2145 (25.91) | 5404 (65.27) |
| Obstetrics and Gynecology Department | 12337(8.23) | 5122526 (12.66) | 70.00 (10.00, 462.00) | 40.00 (19.00, 99.00) | 1450 (11.75) | 3744 (30.35) | 7143 (57.90) |
| Oncology Department | 3951(2.64) | 614002 (1.52) | 13.00 (3.00, 81.50) | 75.00 (30.00, 199.00) | 250 (6.33) | 936 (23.69) | 2765 (69.98) |
| Orthopedics Department | 11340(7.57) | 2650144 (6.55) | 26.00 (5.00, 179.00) | 35.00 (10.00, 99.00) | 1031 (9.09) | 3318 (29.26) | 6991 (61.65) |
| Other Department | 8382(5.59) | 1132566 (2.80) | 10.00 (2.00, 63.00) | 35.00 (9.00, 100.00) | 1315 (15.69) | 2754 (32.86) | 4313 (51.46) |
| Otorhinolaryngology Department | 13928(9.29) | 3791176 (9.37) | 34.00 (6.00, 223.25) | 39.00 (12.50, 100.00) | 1856 (13.33) | 4455 (31.99) | 7617 (54.69) |
| Pediatrics Department | 13406(8.94) | 5361264 (13.25) | 73.00 (10.00, 450.00) | 60.00 (30.00, 119.00) | 2021 (15.08) | 4379 (32.66) | 7006 (52.26) |
| Plastic and Cosmetic Surgery Department | 3036(2.03) | 1234417 (3.05) | 80.00 (12.00, 464.25) | 37.50 (15.00, 90.00) | 291 (9.58) | 1154 (38.01) | 1591 (52.40) |
| Psychiatry and Psychology Department | 2692(1.80) | 779082 (1.93) | 34.00 (6.00, 229.50) | 85.00 (30.00, 200.00) | 356 (13.22) | 821 (30.50) | 1515 (56.28) |
| Pulmonology Department | 2781(1.86) | 565231 (1.40) | 17.00 (3.00, 109.00) | 35.00 (9.00, 100.00) | 311 (11.18) | 780 (28.05) | 1690 (60.77) |
| Traditional Chinese Medicine Department | 18832(12.56) | 4677359 (11.56) | 22.00 (4.00, 156.00) | 30.00 (9.00, 70.00) | 2426 (12.88) | 5810 (30.85) | 10596 (56.27) |
| Urology Department | 6394(4.27) | 1862132 (4.60) | 39.00 (7.00, 258.00) | 50.00 (22.50, 100.00) | 659 (10.31) | 1908 (29.84) | 3827 (59.85) |

### Table S4: The number and proportions of doctors and consultations categorized by healthcare institutions and professional titles

| **Healthcare institution** | **Professional title** | **Number of doctors providing online consultations, *n* (*%*)** | **Number of consultations, *n* (*%*)** |
| --- | --- | --- | --- |
| **Primary hospital** | Primary title | 188 (0.13) | 22972 (0.06) |
|  | Intermediate title | 380 (0.25) | 78151 (0.19) |
|  | Senior title | 234 (0.16) | 73525 (0.18) |
| **Secondary hospital** | Primary title | 2909 (1.94) | 239243 (0.59) |
|  | Intermediate title | 5731 (3.82) | 731789 (1.81) |
|  | Senior title | 5960 (3.98) | 1118371 (2.76) |
| **Tertiary hospital** | Primary title | 14219 (9.49) | 1067574 (2.64) |
|  | Intermediate title | 38319 (25.56) | 7243804 (17.90) |
|  | Senior title | 79640 (53.13) | 28906368 (71.44) |
| **Unclassified hospital** | Primary title | 11 (0.01) | 2472 (0.01) |
|  | Intermediate title | 43 (0.03) | 4213 (0.01) |
|  | Senior title | 59 (0.04) | 25543 (0.06) |
| **Other healthcare institution** | Primary title | 246 (0.16) | 29124 (0.07) |
|  | Intermediate title | 721 (0.48) | 184640 (0.46) |
|  | Senior title | 1230 (0.82) | 735012 (1.82) |

### Table S5: The number and proportions of online consultations provided from 2008 to 2022

| **Number of consultations from 2008 to 2022, n (%)** | | | | | | | | | | | | | | | | |
| --- | --- | --- | --- | --- | --- | --- | --- | --- | --- | --- | --- | --- | --- | --- | --- | --- |
| **Group** | **2008** | **2009** | **2010** | **2011** | **2012** | **2013** | **2014** | **2015** | **2016** | **2017** | **2018** | **2019** | **2020** | **2021** | **2022** | **Total** |
| **Professional title** |  |  |  |  |  |  |  |  |  |  |  |  |  |  |  |  |
| Primary title | 161 (0.59) | 573 (0.22) | 1590 (0.30) | 4337 (0.36) | 6624 (0.44) | 5324 (0.51) | 6864 (0.74) | 89997 (4.64) | 397390 (6.34) | 151210 (3.30) | 77303 (2.12) | 63025 (1.93) | 322515 (6.13) | 106232 (2.30) | 128240 (2.41) | 1361385 (3.36) |
| Intermediate title | 1261 (4.59) | 10890 (4.21) | 26725 (5.02) | 69208 (5.71) | 87418 (5.76) | 69414 (6.63) | 78688 (8.48) | 437291 (22.52) | 1638362 (26.12) | 933505 (20.36) | 701526 (19.21) | 634019 (19.37) | 1496899 (28.45) | 985798 (21.30) | 1071593 (20.12) | 8242597 (20.37) |
| Senior title | 26071 (94.83) | 247097 (95.57) | 503587 (94.68) | 1138025 (93.93) | 1424213 (93.81) | 972620 (92.86) | 841981 (90.78) | 1414210 (72.84) | 4237151 (67.55) | 3500911 (76.35) | 2873086 (78.67) | 2576099 (78.70) | 3441530 (65.42) | 3535143 (76.40) | 4127095 (77.48) | 30858819 (76.26) |
| **Province** |  |  |  |  |  |  |  |  |  |  |  |  |  |  |  |  |
| Anhui | 391 (1.42) | 4309 (1.67) | 8949 (1.68) | 24657 (2.04) | 26746 (1.76) | 17031 (1.63) | 19394 (2.09) | 62698 (3.23) | 282799 (4.51) | 162511 (3.54) | 103233 (2.83) | 78551 (2.40) | 160974 (3.06) | 116578 (2.52) | 130963 (2.46) | 1199784 (2.97) |
| Beijing | 6676 (24.28) | 51995 (20.11) | 99774 (18.76) | 193767 (15.99) | 239709 (15.79) | 172534 (16.47) | 190036 (20.49) | 301838 (15.55) | 515435 (8.22) | 415153 (9.05) | 419349 (11.48) | 444215 (13.57) | 558566 (10.62) | 762622 (16.48) | 1005375 (18.87) | 5377044 (13.29) |
| Chongqing | 365 (1.33) | 2569 (0.99) | 6336 (1.19) | 11208 (0.93) | 18845 (1.24) | 10950 (1.05) | 9138 (0.99) | 21004 (1.08) | 93227 (1.49) | 73252 (1.60) | 48987 (1.34) | 47137 (1.44) | 72480 (1.38) | 69522 (1.50) | 76705 (1.44) | 561725 (1.39) |
| Fujian | 624 (2.27) | 5080 (1.96) | 9804 (1.84) | 22741 (1.88) | 25355 (1.67) | 17248 (1.65) | 13320 (1.44) | 29111 (1.50) | 139420 (2.22) | 98755 (2.15) | 68353 (1.87) | 64906 (1.98) | 137099 (2.61) | 86039 (1.86) | 103681 (1.95) | 821536 (2.03) |
| Gansu | 52 (0.19) | 570 (0.22) | 2311 (0.43) | 5533 (0.46) | 6271 (0.41) | 3132 (0.30) | 1815 (0.20) | 8777 (0.45) | 51509 (0.82) | 45321 (0.99) | 28158 (0.77) | 26251 (0.80) | 45360 (0.86) | 34642 (0.75) | 39304 (0.74) | 299006 (0.74) |
| Guangdong | 2801 (10.19) | 36346 (14.06) | 61470 (11.56) | 121297 (10.01) | 144845 (9.54) | 93806 (8.96) | 82664 (8.91) | 222377 (11.45) | 621035 (9.90) | 451327 (9.84) | 386187 (10.57) | 336169 (10.27) | 434428 (8.26) | 418011 (9.03) | 442193 (8.30) | 3854956 (9.53) |
| Guangxi | 104 (0.38) | 854 (0.33) | 2476 (0.47) | 4674 (0.39) | 4640 (0.31) | 3087 (0.29) | 3405 (0.37) | 7095 (0.37) | 43467 (0.69) | 33234 (0.72) | 26879 (0.74) | 21094 (0.64) | 35455 (0.67) | 21317 (0.46) | 28591 (0.54) | 236372 (0.58) |
| Guizhou | 27 (0.10) | 406 (0.16) | 675 (0.13) | 1963 (0.16) | 3431 (0.23) | 1393 (0.13) | 1655 (0.18) | 3964 (0.20) | 32189 (0.51) | 25011 (0.55) | 21277 (0.58) | 18843 (0.58) | 40670 (0.77) | 28755 (0.62) | 29041 (0.55) | 209300 (0.52) |
| Hainan | 50 (0.18) | 689 (0.27) | 510 (0.10) | 3968 (0.33) | 7679 (0.51) | 4402 (0.42) | 2889 (0.31) | 4170 (0.21) | 16701 (0.27) | 13932 (0.30) | 9975 (0.27) | 7197 (0.22) | 21280 (0.40) | 10035 (0.22) | 10854 (0.20) | 114331 (0.28) |
| Hebei | 842 (3.06) | 6668 (2.58) | 17809 (3.35) | 37998 (3.14) | 38667 (2.55) | 25306 (2.42) | 25775 (2.78) | 87602 (4.51) | 333628 (5.32) | 170256 (3.71) | 98981 (2.71) | 71116 (2.17) | 218883 (4.16) | 123766 (2.67) | 137639 (2.58) | 1394936 (3.45) |
| Heilongjiang | 303 (1.10) | 6754 (2.61) | 10220 (1.92) | 19991 (1.65) | 29263 (1.93) | 18273 (1.74) | 12695 (1.37) | 19191 (0.99) | 90937 (1.45) | 72073 (1.57) | 48329 (1.32) | 48109 (1.47) | 85918 (1.63) | 71288 (1.54) | 84153 (1.58) | 617497 (1.53) |
| Henan | 1303 (4.74) | 13104 (5.07) | 26642 (5.01) | 65176 (5.38) | 71440 (4.71) | 60955 (5.82) | 58466 (6.30) | 102089 (5.26) | 380533 (6.07) | 258299 (5.63) | 172249 (4.72) | 140962 (4.31) | 314294 (5.97) | 177597 (3.84) | 193338 (3.63) | 2036447 (5.03) |
| Hubei | 832 (3.03) | 9740 (3.77) | 23528 (4.42) | 46945 (3.87) | 62626 (4.12) | 40349 (3.85) | 36743 (3.96) | 64783 (3.34) | 252330 (4.02) | 201539 (4.40) | 153284 (4.20) | 141800 (4.33) | 189424 (3.60) | 155352 (3.36) | 152956 (2.87) | 1532231 (3.79) |
| Hunan | 458 (1.67) | 6018 (2.33) | 11120 (2.09) | 27122 (2.24) | 28477 (1.88) | 21462 (2.05) | 19558 (2.11) | 41230 (2.12) | 226639 (3.61) | 154471 (3.37) | 130207 (3.57) | 101183 (3.09) | 167312 (3.18) | 126469 (2.73) | 116771 (2.19) | 1178497 (2.91) |
| Jiangsu | 2116 (7.70) | 22224 (8.60) | 41953 (7.89) | 113786 (9.39) | 132000 (8.69) | 85961 (8.21) | 65090 (7.02) | 111342 (5.73) | 410125 (6.54) | 329457 (7.18) | 246342 (6.75) | 225246 (6.88) | 348534 (6.62) | 282487 (6.10) | 339991 (6.38) | 2756654 (6.81) |
| Jiangxi | 220 (0.80) | 1554 (0.60) | 3901 (0.73) | 9203 (0.76) | 10563 (0.70) | 9507 (0.91) | 7118 (0.77) | 30706 (1.58) | 137840 (2.20) | 85169 (1.86) | 60083 (1.65) | 48220 (1.47) | 91434 (1.74) | 56491 (1.22) | 53433 (1.00) | 605442 (1.50) |
| Jilin | 248 (0.90) | 2411 (0.93) | 4458 (0.84) | 8651 (0.71) | 15328 (1.01) | 8507 (0.81) | 6684 (0.72) | 13542 (0.70) | 57570 (0.92) | 52349 (1.14) | 32210 (0.88) | 28581 (0.87) | 56275 (1.07) | 28925 (0.63) | 37024 (0.70) | 352763 (0.87) |
| Liaoning | 1243 (4.52) | 7839 (3.03) | 17178 (3.23) | 39282 (3.24) | 48882 (3.22) | 31137 (2.97) | 28917 (3.12) | 43293 (2.23) | 182205 (2.90) | 111965 (2.44) | 89991 (2.46) | 84231 (2.57) | 158115 (3.01) | 140911 (3.05) | 149610 (2.81) | 1134799 (2.80) |
| Neimenggu | 6 (0.02) | 678 (0.26) | 1174 (0.22) | 2292 (0.19) | 3264 (0.21) | 2144 (0.20) | 1143 (0.12) | 5285 (0.27) | 53361 (0.85) | 39780 (0.87) | 23435 (0.64) | 16048 (0.49) | 57799 (1.10) | 29459 (0.64) | 27700 (0.52) | 263568 (0.65) |
| Ningxia | 28 (0.10) | 89 (0.03) | 160 (0.03) | 864 (0.07) | 1252 (0.08) | 873 (0.08) | 352 (0.04) | 935 (0.05) | 9216 (0.15) | 33204 (0.72) | 31395 (0.86) | 22909 (0.70) | 30603 (0.58) | 16935 (0.37) | 16226 (0.30) | 165041 (0.41) |
| Qinghai | 4 (0.01) | 122 (0.05) | 70 (0.01) | 666 (0.05) | 394 (0.03) | 161 (0.02) | 76 (0.01) | 52 (0.00) | 2957 (0.05) | 1738 (0.04) | 1121 (0.03) | 910 (0.03) | 1038 (0.02) | 977 (0.02) | 781 (0.01) | 11067 (0.03) |
| Shaanxi | 596 (2.17) | 6211 (2.40) | 14665 (2.76) | 33906 (2.80) | 43709 (2.88) | 33466 (3.20) | 27634 (2.98) | 52111 (2.68) | 220816 (3.52) | 167076 (3.64) | 125209 (3.43) | 116822 (3.57) | 175871 (3.34) | 154710 (3.34) | 171321 (3.22) | 1344123 (3.32) |
| Shandong | 2763 (10.05) | 19104 (7.39) | 43146 (8.11) | 108212 (8.93) | 151490 (9.98) | 101781 (9.72) | 74134 (7.99) | 230063 (11.85) | 648006 (10.33) | 410551 (8.95) | 290979 (7.97) | 214838 (6.56) | 479514 (9.11) | 299569 (6.47) | 354526 (6.66) | 3428676 (8.47) |
| Shanghai | 2578 (9.38) | 28598 (11.06) | 62894 (11.82) | 148883 (12.29) | 182101 (11.99) | 135848 (12.97) | 128632 (13.87) | 191521 (9.86) | 440176 (7.02) | 412155 (8.99) | 433700 (11.88) | 450333 (13.76) | 581403 (11.05) | 808616 (17.48) | 961510 (18.05) | 4968948 (12.28) |
| Shanxi | 261 (0.95) | 2759 (1.07) | 6693 (1.26) | 14858 (1.23) | 23546 (1.55) | 15140 (1.45) | 15424 (1.66) | 55850 (2.88) | 179105 (2.86) | 101805 (2.22) | 58349 (1.60) | 46167 (1.41) | 71063 (1.35) | 40322 (0.87) | 40957 (0.77) | 672299 (1.66) |
| Sichuan | 633 (2.30) | 5077 (1.96) | 15910 (2.99) | 35330 (2.92) | 43758 (2.88) | 31066 (2.97) | 30918 (3.33) | 56835 (2.93) | 233892 (3.73) | 182166 (3.97) | 133433 (3.65) | 106294 (3.25) | 149201 (2.84) | 105769 (2.29) | 111498 (2.09) | 1241780 (3.07) |
| Tianjin | 522 (1.90) | 5675 (2.19) | 16667 (3.13) | 46063 (3.80) | 62284 (4.10) | 37853 (3.61) | 17557 (1.89) | 49603 (2.55) | 117984 (1.88) | 90259 (1.97) | 86615 (2.37) | 83896 (2.56) | 127575 (2.42) | 122082 (2.64) | 127336 (2.39) | 991971 (2.45) |
| Xinjiang | 33 (0.12) | 64 (0.02) | 162 (0.03) | 880 (0.07) | 2212 (0.15) | 1444 (0.14) | 768 (0.08) | 2560 (0.13) | 18957 (0.30) | 17715 (0.39) | 16455 (0.45) | 13716 (0.42) | 35985 (0.68) | 21846 (0.47) | 30035 (0.56) | 162832 (0.40) |
| Xizang |  |  |  |  |  | 27 (0.00) | 2 (0.00) |  | 29 (0.00) | 783 (0.02) | 1107 (0.03) | 453 (0.01) | 1706 (0.03) | 430 (0.01) | 1217 (0.02) | 5754 (0.01) |
| Yunnan | 48 (0.17) | 792 (0.31) | 2786 (0.52) | 6184 (0.51) | 8538 (0.56) | 7997 (0.76) | 7295 (0.79) | 8224 (0.42) | 36181 (0.58) | 52128 (1.14) | 45298 (1.24) | 39127 (1.20) | 54030 (1.03) | 41020 (0.89) | 42833 (0.80) | 352481 (0.87) |
| Zhejiang | 1366 (4.97) | 10261 (3.97) | 18461 (3.47) | 55470 (4.58) | 80940 (5.33) | 54518 (5.21) | 38236 (4.12) | 113647 (5.85) | 444634 (7.09) | 322192 (7.03) | 260745 (7.14) | 227819 (6.96) | 358655 (6.82) | 274631 (5.94) | 309366 (5.81) | 2570941 (6.35) |
| **Department** |  |  |  |  |  |  |  |  |  |  |  |  |  |  |  |  |
| Cardiology Department | 1413 (5.14) | 13926 (5.39) | 27484 (5.17) | 58790 (4.85) | 56717 (3.74) | 31332 (2.99) | 33551 (3.62) | 62690 (3.23) | 154880 (2.47) | 161657 (3.53) | 140615 (3.85) | 127217 (3.89) | 254594 (4.84) | 151571 (3.28) | 177114 (3.32) | 1453551 (3.59) |
| Dermatology and Venereology Department | 1059 (3.85) | 6031 (2.33) | 10415 (1.96) | 28356 (2.34) | 44479 (2.93) | 37136 (3.55) | 35829 (3.86) | 91206 (4.70) | 364792 (5.82) | 251319 (5.48) | 183494 (5.02) | 160736 (4.91) | 295737 (5.62) | 280437 (6.06) | 320973 (6.03) | 2111999 (5.22) |
| Endocrinology Department | 413 (1.50) | 5523 (2.14) | 8737 (1.64) | 23119 (1.91) | 32454 (2.14) | 20259 (1.93) | 23913 (2.58) | 48228 (2.48) | 111646 (1.78) | 100049 (2.18) | 87168 (2.39) | 70201 (2.14) | 164862 (3.13) | 115905 (2.50) | 141487 (2.66) | 953964 (2.36) |
| Family Medicine Department | 203 (0.74) | 1342 (0.52) | 2039 (0.38) | 3749 (0.31) | 4633 (0.31) | 3088 (0.29) | 4550 (0.49) | 17029 (0.88) | 95329 (1.52) | 55340 (1.21) | 24134 (0.66) | 18266 (0.56) | 156080 (2.97) | 28812 (0.62) | 61964 (1.16) | 476558 (1.18) |
| Gastroenterology Department | 1545 (5.62) | 16378 (6.33) | 32990 (6.20) | 64460 (5.32) | 77448 (5.10) | 48071 (4.59) | 36097 (3.89) | 75378 (3.88) | 218480 (3.48) | 191185 (4.17) | 174488 (4.78) | 156125 (4.77) | 239687 (4.56) | 238851 (5.16) | 269480 (5.06) | 1840663 (4.55) |
| General Surgery Department | 1778 (6.47) | 19502 (7.54) | 46438 (8.73) | 97464 (8.04) | 108167 (7.12) | 70337 (6.72) | 53563 (5.77) | 136332 (7.02) | 387087 (6.17) | 318135 (6.94) | 287058 (7.86) | 270515 (8.26) | 324736 (6.17) | 429423 (9.28) | 492928 (9.25) | 3043463 (7.52) |
| Infectious Diseases Department | 500 (1.82) | 4938 (1.91) | 8711 (1.64) | 17294 (1.43) | 21089 (1.39) | 14841 (1.42) | 14360 (1.55) | 29558 (1.52) | 62846 (1.00) | 47719 (1.04) | 39060 (1.07) | 41399 (1.26) | 85848 (1.63) | 48676 (1.05) | 58725 (1.10) | 495564 (1.22) |
| Medical Technology Department | 275 (1.00) | 4236 (1.64) | 12838 (2.41) | 21091 (1.74) | 21495 (1.42) | 15461 (1.48) | 9366 (1.01) | 21826 (1.12) | 76167 (1.21) | 58909 (1.28) | 48318 (1.32) | 43819 (1.34) | 61183 (1.16) | 82052 (1.77) | 98046 (1.84) | 575082 (1.42) |
| Neurology Department | 1819 (6.62) | 17667 (6.83) | 33985 (6.39) | 71163 (5.87) | 84830 (5.59) | 52021 (4.97) | 46339 (5.00) | 80225 (4.13) | 194744 (3.10) | 171670 (3.74) | 151826 (4.16) | 134657 (4.11) | 237217 (4.51) | 203907 (4.41) | 239988 (4.51) | 1722058 (4.26) |
| Obstetrics and Gynecology Department | 1860 (6.77) | 12101 (4.68) | 36320 (6.83) | 99332 (8.20) | 137924 (9.08) | 102182 (9.76) | 121027 (13.05) | 259759 (13.38) | 997146 (15.90) | 634941 (13.85) | 501682 (13.74) | 438075 (13.38) | 584262 (11.11) | 560662 (12.12) | 635253 (11.93) | 5122526 (12.66) |
| Oncology Department | 428 (1.56) | 8221 (3.18) | 14937 (2.81) | 20520 (1.69) | 21187 (1.40) | 15445 (1.47) | 11599 (1.25) | 21753 (1.12) | 54183 (0.86) | 50175 (1.09) | 59468 (1.63) | 51048 (1.56) | 74855 (1.42) | 89228 (1.93) | 120955 (2.27) | 614002 (1.52) |
| Orthopedics Department | 2250 (8.18) | 21932 (8.48) | 48798 (9.17) | 104737 (8.64) | 121440 (8.00) | 84689 (8.09) | 75995 (8.19) | 152633 (7.86) | 385793 (6.15) | 295876 (6.45) | 240029 (6.57) | 219414 (6.70) | 280394 (5.33) | 299667 (6.48) | 316497 (5.94) | 2650144 (6.55) |
| Otorhinolaryngology Department | 2885 (10.49) | 25806 (9.98) | 47898 (9.01) | 121715 (10.05) | 155465 (10.24) | 107739 (10.29) | 83948 (9.05) | 177110 (9.12) | 640877 (10.22) | 448477 (9.78) | 347771 (9.52) | 309656 (9.46) | 439034 (8.35) | 420269 (9.08) | 462526 (8.68) | 3791176 (9.37) |
| Pediatrics Department | 2042 (7.43) | 16708 (6.46) | 39513 (7.43) | 102312 (8.44) | 136116 (8.97) | 89475 (8.54) | 86119 (9.28) | 223346 (11.50) | 975084 (15.54) | 627563 (13.69) | 479939 (13.14) | 430136 (13.14) | 675492 (12.84) | 652702 (14.11) | 824717 (15.48) | 5361264 (13.25) |
| Plastic and Cosmetic Surgery Department | 497 (1.81) | 8925 (3.45) | 18792 (3.53) | 36470 (3.01) | 43679 (2.88) | 37969 (3.63) | 35538 (3.83) | 50598 (2.61) | 161284 (2.57) | 137676 (3.00) | 110554 (3.03) | 130563 (3.99) | 152719 (2.90) | 162292 (3.51) | 146861 (2.76) | 1234417 (3.05) |
| Psychiatry and Psychology Department | 604 (2.20) | 5748 (2.22) | 10674 (2.01) | 26075 (2.15) | 31045 (2.04) | 23214 (2.22) | 16016 (1.73) | 25928 (1.34) | 103217 (1.65) | 77319 (1.69) | 63678 (1.74) | 63963 (1.95) | 112024 (2.13) | 109296 (2.36) | 110281 (2.07) | 779082 (1.93) |
| Pulmonology Department | 487 (1.77) | 3810 (1.47) | 8352 (1.57) | 22939 (1.89) | 24174 (1.59) | 12339 (1.18) | 10527 (1.13) | 22984 (1.18) | 61227 (0.98) | 41767 (0.91) | 41307 (1.13) | 38427 (1.17) | 143411 (2.73) | 44765 (0.97) | 88715 (1.67) | 565231 (1.40) |
| Traditional Chinese Medicine Department | 5226 (19.01) | 46670 (18.05) | 86937 (16.34) | 213342 (17.61) | 304969 (20.09) | 225171 (21.50) | 176447 (19.02) | 301446 (15.53) | 800248 (12.76) | 584802 (12.75) | 389074 (10.65) | 308671 (9.43) | 534072 (10.15) | 346403 (7.49) | 353881 (6.64) | 4677359 (11.56) |
| Urology Department | 1411 (5.13) | 11668 (4.51) | 19362 (3.64) | 45089 (3.72) | 49904 (3.29) | 31830 (3.04) | 31912 (3.44) | 89899 (4.63) | 270809 (4.32) | 228108 (4.97) | 195860 (5.36) | 186980 (5.71) | 221912 (4.22) | 233298 (5.04) | 244090 (4.58) | 1862132 (4.60) |
| Other Department | 798 (2.90) | 7428 (2.87) | 16682 (3.14) | 33553 (2.77) | 41040 (2.70) | 24759 (2.36) | 20837 (2.25) | 53570 (2.76) | 157064 (2.50) | 102939 (2.24) | 86392 (2.37) | 73275 (2.24) | 222825 (4.24) | 128957 (2.79) | 162447 (3.05) | 1132566 (2.80) |
| Total | 27493 | 258560 | 531902 | 1211570 | 1518255 | 1047358 | 927533 | 1941498 | 6272903 | 4585626 | 3651915 | 3273143 | 5260944 | 4627173 | 5326928 | 40462801 |

### Table S6: The number of consultations provided per active doctor from 2008 to 2022

| **Group** | **Number of consultations provided per active doctor from 2008 to 2022** | | | | | | | | | | | | | | | |
| --- | --- | --- | --- | --- | --- | --- | --- | --- | --- | --- | --- | --- | --- | --- | --- | --- |
|  | **2008** | **2009** | **2010** | **2011** | **2012** | **2013** | **2014** | **2015** | **2016** | **2017** | **2018** | **2019** | **2020** | **2021** | **2022** | **Total** |
| **Professional title** |  |  |  |  |  |  |  |  |  |  |  |  |  |  |  |  |
| Primary title | 2.00 (1.00, 4.00) | 2.00 (1.00, 8.25) | 4.50 (1.75, 13.00) | 6.00 (2.00, 18.00) | 6.00 (2.00, 18.75) | 5.50 (2.00, 18.75) | 4.00 (2.00, 15.75) | 9.00 (2.00, 55.50) | 14.00 (3.00, 78.00) | 10.00 (3.00, 35.00) | 5.00 (2.00, 16.00) | 4.00 (1.00, 13.00) | 5.00 (1.00, 18.00) | 4.00 (1.00, 14.00) | 3.00 (1.00, 11.00) | 8.00 (2.00, 35.00) |
| Intermediate title | 2.00 (1.00, 5.50) | 5.00 (2.00, 18.25) | 6.00 (2.00, 25.00) | 12.00 (3.00, 50.00) | 10.00 (3.00, 44.00) | 8.00 (2.00, 32.00) | 7.00 (2.00, 29.00) | 11.00 (2.00, 64.00) | 20.00 (4.00, 113.50) | 16.00 (4.00, 67.00) | 9.00 (2.00, 38.00) | 8.00 (2.00, 32.00) | 8.00 (2.00, 35.00) | 7.00 (2.00, 31.00) | 5.00 (2.00, 25.00) | 19.00 (4.00, 111.00) |
| Senior title | 4.00 (2.00, 11.00) | 8.00 (2.00, 29.00) | 11.00 (3.00, 46.00) | 20.00 (4.00, 92.00) | 22.00 (4.00, 103.25) | 15.00 (3.00, 66.00) | 10.00 (2.00, 54.75) | 12.00 (3.00, 54.00) | 21.00 (4.00, 110.00) | 29.00 (6.00, 107.00) | 19.00 (4.00, 75.00) | 17.00 (4.00, 69.00) | 15.00 (4.00, 62.00) | 13.00 (3.00, 65.00) | 9.00 (2.00, 46.00) | 52.00 (7.00, 349.00) |
| **Province** |  |  |  |  |  |  |  |  |  |  |  |  |  |  |  |  |
| Anhui | 3.00 (1.00, 6.50) | 7.00 (2.00, 23.50) | 7.00 (2.00, 29.00) | 13.00 (2.00, 62.00) | 13.00 (3.00, 72.00) | 10.00 (3.00, 36.25) | 7.00 (2.00, 41.50) | 12.00 (3.00, 54.00) | 28.00 (5.00, 133.00) | 23.00 (5.00, 95.00) | 12.00 (3.00, 56.00) | 11.00 (3.00, 45.00) | 10.00 (3.00, 51.00) | 7.00 (2.00, 35.00) | 5.00 (2.00, 25.00) | 26.00 (5.00, 186.00) |
| Beijing | 5.00 (2.00, 15.00) | 10.00 (3.00, 38.00) | 13.00 (3.00, 62.00) | 23.00 (4.00, 117.00) | 24.00 (5.00, 118.00) | 18.00 (3.00, 79.00) | 14.00 (3.00, 80.00) | 16.00 (3.00, 75.00) | 17.00 (3.00, 94.00) | 25.00 (5.00, 93.00) | 20.00 (5.00, 77.00) | 21.00 (5.00, 81.00) | 20.00 (5.00, 76.00) | 23.50 (5.00, 101.75) | 18.00 (4.00, 95.00) | 77.00 (10.00, 469.00) |
| Chongqing | 4.00 (3.00, 10.25) | 5.00 (2.00, 19.50) | 7.50 (3.00, 23.25) | 13.50 (3.00, 56.25) | 13.50 (4.00, 76.75) | 14.00 (4.00, 67.50) | 13.50 (2.00, 67.00) | 7.00 (2.00, 41.00) | 19.50 (4.00, 103.00) | 21.00 (5.00, 101.50) | 14.00 (3.00, 56.00) | 13.00 (3.00, 53.00) | 10.00 (2.00, 39.00) | 10.00 (2.00, 48.00) | 7.00 (2.00, 37.00) | 21.00 (4.00, 158.00) |
| Fujian | 5.00 (1.00, 12.00) | 8.00 (2.00, 27.00) | 11.00 (3.00, 44.50) | 24.00 (6.00, 86.50) | 15.00 (3.00, 65.00) | 12.00 (3.00, 51.00) | 9.00 (2.00, 44.00) | 8.00 (2.00, 42.00) | 18.50 (4.00, 96.75) | 22.00 (5.00, 83.75) | 11.00 (3.00, 46.00) | 10.00 (3.00, 39.00) | 9.00 (2.00, 40.00) | 7.00 (2.00, 32.00) | 5.00 (1.00, 22.00) | 20.00 (4.00, 138.00) |
| Gansu | 3.00 (1.50, 5.00) | 5.00 (2.00, 14.50) | 6.00 (2.00, 15.75) | 11.00 (3.00, 46.00) | 11.00 (4.00, 58.25) | 6.00 (2.00, 35.00) | 6.00 (2.00, 23.00) | 7.00 (2.00, 26.25) | 22.50 (4.00, 108.00) | 22.00 (5.00, 83.00) | 11.00 (3.00, 40.25) | 12.00 (3.00, 41.75) | 9.00 (3.00, 40.00) | 7.00 (2.00, 29.00) | 5.00 (2.00, 25.00) | 19.00 (3.00, 128.00) |
| Guangdong | 4.00 (2.00, 11.00) | 9.00 (2.00, 35.00) | 12.00 (3.00, 52.50) | 22.00 (4.00, 103.00) | 22.00 (4.00, 102.00) | 16.00 (3.00, 62.25) | 10.00 (2.00, 50.00) | 13.00 (3.00, 61.00) | 23.00 (4.00, 115.00) | 23.00 (5.00, 100.00) | 15.00 (4.00, 67.00) | 13.00 (3.00, 56.00) | 11.00 (3.00, 47.00) | 10.00 (2.00, 50.00) | 6.00 (2.00, 34.00) | 30.00 (5.00, 213.00) |
| Guangxi | 3.00 (1.00, 4.50) | 3.00 (2.00, 21.00) | 9.00 (2.00, 56.00) | 11.00 (3.75, 70.50) | 9.00 (3.00, 52.75) | 12.00 (2.00, 38.50) | 5.00 (2.75, 23.50) | 5.00 (2.00, 25.75) | 18.50 (3.00, 103.50) | 32.00 (6.00, 115.00) | 14.00 (4.00, 50.50) | 11.00 (3.00, 39.00) | 8.00 (2.00, 29.00) | 5.00 (2.00, 19.75) | 4.00 (1.00, 16.00) | 15.00 (3.00, 87.00) |
| Guizhou | 2.00 (1.00, 6.00) | 7.00 (4.50, 33.50) | 6.00 (2.00, 14.50) | 24.00 (5.00, 46.00) | 12.50 (3.00, 35.75) | 8.00 (3.00, 29.00) | 4.00 (2.00, 19.00) | 9.00 (2.00, 24.00) | 23.00 (5.00, 95.50) | 24.00 (5.00, 77.75) | 12.00 (3.00, 39.75) | 10.00 (3.00, 37.00) | 8.00 (2.00, 34.75) | 7.00 (2.00, 28.50) | 4.00 (1.00, 21.00) | 18.00 (4.00, 95.00) |
| Hainan | 4.00 (1.50, 7.50) | 4.50 (2.25, 11.50) | 7.00 (3.00, 14.50) | 14.50 (5.25, 81.50) | 19.50 (5.75, 73.75) | 14.00 (3.00, 36.50) | 7.00 (3.00, 58.00) | 7.00 (2.00, 43.50) | 19.50 (3.00, 111.75) | 21.00 (5.00, 88.00) | 15.00 (4.00, 46.00) | 12.50 (3.00, 36.75) | 7.00 (2.00, 30.25) | 6.00 (2.00, 31.00) | 5.00 (1.00, 20.00) | 13.00 (3.00, 96.50) |
| Hebei | 3.00 (2.00, 8.00) | 5.00 (2.00, 15.00) | 8.00 (2.00, 28.00) | 12.00 (3.00, 62.25) | 12.00 (3.00, 58.25) | 9.00 (2.00, 42.00) | 6.00 (2.00, 34.00) | 10.00 (2.00, 56.25) | 21.00 (4.00, 120.00) | 23.00 (5.00, 95.25) | 11.00 (3.00, 42.00) | 9.00 (3.00, 34.00) | 8.00 (2.00, 39.00) | 6.00 (2.00, 25.00) | 5.00 (2.00, 19.00) | 20.00 (4.00, 141.00) |
| Heilongjiang | 2.50 (2.00, 6.25) | 6.00 (2.00, 14.25) | 10.00 (3.00, 42.00) | 15.50 (3.00, 63.50) | 14.00 (4.00, 76.50) | 8.00 (3.00, 38.25) | 9.00 (2.00, 40.00) | 9.50 (2.00, 38.00) | 19.00 (4.00, 100.00) | 23.00 (5.00, 98.00) | 12.00 (3.00, 53.00) | 11.00 (3.00, 49.00) | 10.00 (3.00, 41.75) | 7.00 (2.00, 34.50) | 5.00 (2.00, 22.00) | 19.00 (4.00, 149.00) |
| Henan | 4.00 (2.00, 9.00) | 7.00 (2.00, 25.00) | 8.00 (3.00, 31.00) | 16.00 (3.00, 62.00) | 14.00 (4.00, 64.00) | 11.00 (3.00, 44.00) | 8.00 (2.00, 44.00) | 9.00 (2.00, 46.50) | 19.00 (4.00, 96.00) | 20.00 (4.00, 86.00) | 12.00 (3.00, 53.00) | 10.00 (3.00, 40.00) | 9.50 (2.00, 43.00) | 6.00 (2.00, 29.00) | 4.00 (2.00, 20.00) | 21.00 (4.00, 144.00) |
| Hubei | 3.00 (1.00, 8.50) | 8.00 (2.00, 31.00) | 10.00 (3.00, 45.00) | 16.00 (3.00, 85.00) | 20.00 (4.75, 103.00) | 13.00 (3.00, 59.00) | 10.00 (2.00, 52.00) | 10.00 (2.00, 56.75) | 21.00 (4.00, 112.75) | 24.00 (5.00, 91.00) | 14.50 (4.00, 59.00) | 12.00 (3.00, 51.00) | 10.00 (3.00, 42.00) | 8.00 (2.00, 39.00) | 5.00 (2.00, 27.00) | 25.00 (5.00, 173.00) |
| Hunan | 3.00 (1.00, 12.75) | 5.00 (2.00, 22.00) | 9.00 (3.00, 38.75) | 17.00 (3.00, 82.50) | 17.00 (3.00, 71.50) | 11.00 (3.00, 42.00) | 9.50 (2.00, 42.50) | 10.00 (2.00, 42.00) | 25.00 (5.00, 127.00) | 22.00 (4.00, 89.75) | 14.50 (3.00, 63.00) | 12.00 (3.00, 52.00) | 10.00 (2.00, 47.00) | 8.00 (2.00, 42.00) | 5.00 (2.00, 28.00) | 27.00 (4.00, 168.75) |
| Jiangsu | 4.00 (2.00, 10.00) | 8.00 (3.00, 31.00) | 9.00 (3.00, 49.00) | 25.00 (5.00, 104.00) | 23.00 (5.00, 109.00) | 18.00 (4.00, 76.00) | 11.00 (3.00, 52.00) | 12.00 (3.00, 50.00) | 21.00 (4.00, 103.00) | 24.00 (5.00, 95.00) | 14.00 (3.00, 60.00) | 13.00 (3.00, 52.00) | 10.00 (3.00, 44.00) | 8.00 (2.00, 39.00) | 6.00 (2.00, 30.00) | 30.00 (5.00, 202.00) |
| Jiangxi | 2.00 (1.00, 6.00) | 5.00 (2.00, 13.25) | 9.00 (2.00, 35.25) | 9.00 (2.00, 63.75) | 14.00 (4.00, 42.00) | 10.50 (2.75, 33.25) | 7.00 (2.00, 24.00) | 9.50 (2.00, 52.25) | 27.00 (5.00, 137.00) | 21.00 (4.00, 91.00) | 12.00 (3.00, 52.50) | 9.00 (2.00, 38.25) | 8.00 (2.00, 39.00) | 6.00 (2.00, 26.00) | 4.00 (1.00, 15.00) | 17.00 (3.00, 119.50) |
| Jilin | 2.50 (1.00, 10.00) | 4.50 (1.25, 14.00) | 8.00 (2.00, 30.00) | 7.50 (3.00, 41.75) | 11.00 (3.00, 58.50) | 8.00 (2.00, 37.00) | 6.00 (2.00, 28.00) | 9.00 (2.00, 27.25) | 17.00 (3.00, 89.50) | 21.00 (6.00, 74.00) | 10.50 (2.00, 47.25) | 11.00 (2.00, 36.00) | 8.00 (3.00, 39.00) | 6.00 (2.00, 23.00) | 5.00 (2.00, 19.00) | 15.00 (4.00, 94.00) |
| Liaoning | 4.00 (1.00, 12.25) | 7.00 (2.00, 30.50) | 12.00 (4.00, 39.00) | 17.50 (4.00, 94.00) | 19.00 (4.00, 87.50) | 11.00 (3.00, 55.00) | 9.00 (2.00, 53.00) | 11.00 (3.00, 50.00) | 20.00 (4.00, 112.75) | 22.00 (4.00, 81.00) | 13.00 (3.00, 56.00) | 11.00 (3.00, 49.00) | 10.00 (3.00, 45.00) | 9.00 (2.00, 45.50) | 7.00 (2.00, 36.00) | 25.00 (4.00, 169.00) |
| Neimenggu | 1.00 (1.00, 2.50) | 3.00 (2.00, 10.00) | 3.00 (1.25, 9.50) | 5.00 (2.00, 14.00) | 10.00 (4.00, 27.50) | 4.00 (2.00, 18.00) | 4.00 (1.00, 11.00) | 4.00 (1.00, 21.25) | 23.00 (4.00, 127.50) | 21.00 (4.00, 62.25) | 8.00 (3.00, 38.00) | 8.00 (2.00, 26.00) | 5.00 (2.00, 20.00) | 4.00 (1.00, 14.00) | 3.00 (1.00, 10.00) | 10.00 (3.00, 61.00) |
| Ningxia | 2.00 (1.00, 4.00) | 3.00 (2.00, 7.00) | 4.00 (1.00, 14.00) | 6.00 (2.75, 29.25) | 7.00 (3.00, 27.00) | 6.00 (3.00, 25.00) | 5.00 (2.00, 13.00) | 3.00 (1.00, 9.00) | 10.00 (3.00, 40.00) | 9.00 (2.00, 41.00) | 7.00 (2.00, 29.00) | 6.00 (2.00, 27.00) | 5.00 (1.00, 23.00) | 5.00 (1.00, 22.00) | 3.00 (1.00, 12.00) | 10.00 (2.00, 61.00) |
| Qinghai | 2.00 (1.50, 2.50) | 1.00 (1.00, 4.00) | 5.00 (5.00, 14.00) | 5.00 (1.50, 24.00) | 18.50 (4.00, 35.50) | 4.50 (1.75, 17.75) | 3.00 (1.00, 5.00) | 2.00 (1.00, 3.00) | 17.50 (3.75, 64.00) | 11.00 (2.50, 30.00) | 5.00 (1.75, 14.00) | 5.50 (2.00, 12.50) | 2.50 (1.00, 6.00) | 3.50 (1.00, 7.25) | 3.00 (1.00, 6.00) | 7.00 (2.00, 30.50) |
| Shaanxi | 3.00 (1.00, 8.50) | 8.00 (2.00, 25.75) | 9.00 (2.00, 43.25) | 14.00 (3.00, 69.00) | 16.50 (4.00, 73.50) | 11.00 (2.00, 56.00) | 9.00 (2.00, 41.00) | 11.00 (3.00, 51.00) | 23.00 (5.00, 118.00) | 29.00 (6.00, 112.00) | 18.00 (4.00, 76.00) | 15.00 (3.00, 62.00) | 12.00 (3.00, 56.00) | 10.00 (2.00, 50.00) | 7.00 (2.00, 37.00) | 31.00 (5.00, 232.00) |
| Shandong | 4.00 (2.00, 13.00) | 8.00 (2.00, 25.00) | 9.00 (3.00, 43.00) | 20.00 (4.00, 83.00) | 22.00 (5.00, 106.00) | 17.00 (4.00, 71.00) | 7.00 (2.00, 41.00) | 11.00 (3.00, 63.00) | 18.00 (4.00, 113.00) | 23.00 (5.00, 84.75) | 13.00 (3.00, 51.50) | 10.00 (3.00, 43.00) | 10.00 (3.00, 45.00) | 8.00 (2.00, 35.00) | 6.00 (2.00, 26.00) | 24.00 (4.00, 172.00) |
| Shanghai | 4.50 (2.00, 18.00) | 8.00 (3.00, 34.00) | 15.00 (3.00, 54.00) | 27.00 (4.00, 112.50) | 28.00 (5.00, 122.00) | 18.00 (4.00, 77.00) | 15.00 (3.00, 74.00) | 14.00 (3.00, 60.00) | 18.00 (4.00, 91.00) | 26.00 (5.00, 92.00) | 21.00 (5.00, 80.00) | 20.00 (5.00, 77.00) | 18.00 (4.00, 76.00) | 23.00 (5.00, 99.00) | 16.00 (3.00, 84.00) | 72.00 (10.00, 427.00) |
| Shanxi | 3.00 (1.00, 7.75) | 5.00 (2.00, 24.50) | 7.50 (2.00, 30.25) | 14.00 (3.00, 55.00) | 15.00 (3.00, 58.50) | 10.00 (2.50, 38.50) | 9.00 (2.00, 30.25) | 11.00 (2.00, 59.00) | 21.00 (4.00, 142.00) | 26.50 (6.00, 102.25) | 11.00 (3.00, 55.25) | 9.00 (2.00, 37.00) | 8.00 (2.00, 30.00) | 4.00 (1.00, 18.00) | 3.00 (1.00, 12.00) | 20.00 (4.00, 153.00) |
| Sichuan | 3.00 (1.00, 5.00) | 6.50 (3.00, 21.00) | 9.00 (3.00, 35.00) | 19.00 (4.00, 80.00) | 18.00 (4.00, 93.25) | 12.00 (3.00, 54.25) | 9.00 (2.00, 43.75) | 12.00 (3.00, 54.00) | 21.00 (5.00, 116.75) | 24.00 (5.00, 106.00) | 14.00 (3.00, 58.25) | 11.00 (3.00, 49.00) | 9.00 (2.00, 40.00) | 8.00 (2.00, 32.00) | 5.00 (2.00, 23.00) | 24.00 (4.00, 161.00) |
| Tianjin | 4.50 (2.00, 9.25) | 11.00 (3.00, 31.00) | 12.00 (3.00, 44.00) | 29.50 (7.00, 103.75) | 26.50 (5.00, 127.50) | 18.00 (4.00, 68.00) | 8.00 (3.00, 31.00) | 12.00 (2.00, 51.00) | 16.00 (3.00, 95.00) | 22.00 (5.00, 83.00) | 16.00 (4.00, 59.00) | 14.00 (3.75, 55.00) | 13.00 (3.00, 58.00) | 11.00 (3.00, 48.00) | 7.00 (2.00, 36.00) | 38.00 (6.00, 257.75) |
| Xinjiang | 2.00 (1.00, 5.00) | 2.00 (1.00, 4.00) | 2.00 (1.00, 8.75) | 5.00 (2.00, 25.50) | 5.00 (2.00, 21.00) | 6.00 (2.00, 24.50) | 4.00 (1.00, 11.75) | 6.50 (2.00, 42.50) | 18.00 (3.00, 65.00) | 13.00 (3.00, 51.50) | 9.00 (2.00, 31.00) | 8.00 (2.00, 30.25) | 8.00 (2.00, 27.50) | 5.00 (2.00, 21.00) | 5.00 (2.00, 17.00) | 12.00 (3.00, 54.00) |
| Xizang | - | - | - | - | - | 27.00 (27.00, 27.00) | 1.00 (1.00, 1.00) |  | 8.00 (5.50, 13.00) | 5.00 (1.00, 6.00) | 7.00 (2.00, 23.00) | 6.00 (2.00, 19.00) | 7.00 (5.00, 32.00) | 8.00 (2.00, 13.75) | 3.00 (2.00, 6.00) | 8.00 (3.00, 31.00) |
| Yunnan | 2.00 (1.00, 3.00) | 6.00 (2.00, 15.00) | 9.00 (2.75, 29.25) | 17.00 (3.50, 77.00) | 19.50 (4.00, 88.00) | 14.00 (4.00, 61.00) | 8.00 (2.00, 60.75) | 12.00 (2.00, 42.50) | 21.00 (4.00, 91.75) | 29.50 (6.00, 97.00) | 19.50 (4.00, 63.00) | 14.00 (3.00, 49.00) | 10.00 (3.00, 40.50) | 9.00 (2.00, 35.00) | 6.00 (2.00, 22.00) | 22.00 (4.00, 151.75) |
| Zhejiang | 5.00 (2.00, 12.00) | 8.00 (3.00, 27.50) | 10.00 (3.00, 45.00) | 24.00 (5.00, 101.00) | 29.00 (6.00, 124.25) | 19.00 (4.00, 68.00) | 9.00 (2.00, 44.00) | 12.00 (3.00, 53.00) | 23.00 (4.00, 119.50) | 26.00 (5.00, 101.00) | 16.00 (4.00, 61.00) | 13.00 (3.00, 54.00) | 12.00 (3.00, 50.00) | 10.00 (3.00, 43.00) | 6.00 (2.00, 29.00) | 31.00 (6.00, 216.00) |
| **Department** |  |  |  |  |  |  |  |  |  |  |  |  |  |  |  |  |
| Cardiology Department | 4.00 (1.00, 11.00) | 7.00 (2.00, 25.00) | 9.00 (3.00, 52.00) | 17.00 (3.00, 88.00) | 16.00 (4.00, 75.50) | 10.00 (3.00, 41.25) | 10.00 (2.00, 49.00) | 9.00 (2.00, 40.00) | 14.00 (3.00, 55.00) | 16.00 (4.00, 62.00) | 11.00 (3.00, 43.00) | 9.00 (2.00, 36.00) | 9.00 (2.00, 35.00) | 7.00 (2.00, 39.00) | 5.00 (2.00, 28.00) | 19.00 (4.00, 102.00) |
| Dermatology and Venereology Department | 7.00 (2.00, 17.00) | 17.00 (4.00, 46.00) | 14.00 (3.00, 50.00) | 26.00 (5.00, 124.00) | 30.00 (5.00, 142.50) | 25.00 (4.75, 109.50) | 15.00 (3.00, 87.00) | 25.00 (4.00, 114.00) | 44.50 (7.00, 262.50) | 37.00 (9.00, 131.00) | 24.00 (6.00, 90.00) | 21.00 (6.00, 76.00) | 21.00 (5.00, 87.00) | 16.00 (4.00, 71.00) | 11.50 (3.00, 60.00) | 78.00 (12.00, 511.00) |
| Endocrinology Department | 3.00 (1.00, 8.00) | 6.00 (2.75, 22.00) | 8.00 (2.00, 29.00) | 17.00 (3.00, 86.50) | 18.50 (4.00, 102.00) | 14.00 (3.00, 70.00) | 8.00 (2.00, 38.25) | 11.50 (3.00, 53.75) | 15.00 (3.00, 78.00) | 20.00 (5.00, 86.25) | 14.00 (4.00, 59.00) | 12.00 (3.00, 48.00) | 10.00 (2.00, 54.00) | 10.00 (3.00, 50.00) | 8.00 (2.00, 45.00) | 23.00 (5.00, 166.25) |
| Family Medicine Department | 4.00 (3.00, 8.00) | 5.00 (1.00, 16.00) | 5.00 (2.00, 27.50) | 9.00 (2.00, 40.00) | 11.50 (2.00, 40.75) | 7.00 (2.00, 48.00) | 6.50 (2.00, 25.50) | 7.00 (2.00, 53.75) | 13.00 (3.00, 89.00) | 12.00 (3.00, 50.00) | 6.00 (2.00, 28.00) | 7.00 (2.00, 27.00) | 8.00 (2.00, 63.00) | 4.00 (2.00, 16.00) | 4.00 (1.00, 19.00) | 12.00 (3.00, 72.00) |
| Gastroenterology Department | 5.00 (2.00, 13.25) | 8.00 (3.00, 30.00) | 8.00 (2.00, 39.00) | 19.00 (4.00, 73.00) | 18.50 (4.00, 79.25) | 15.00 (3.00, 49.00) | 8.00 (2.00, 33.00) | 8.00 (2.00, 35.00) | 14.00 (3.00, 69.00) | 19.00 (4.00, 72.00) | 12.00 (3.00, 51.00) | 12.00 (3.00, 45.00) | 10.00 (3.00, 43.50) | 10.00 (2.00, 45.00) | 6.00 (2.00, 31.00) | 26.00 (5.00, 174.00) |
| General Surgery Department | 3.00 (1.00, 7.00) | 7.00 (2.00, 23.00) | 8.00 (2.00, 37.00) | 15.00 (3.00, 64.00) | 16.00 (4.00, 73.00) | 11.00 (3.00, 43.00) | 7.00 (2.00, 32.50) | 9.00 (2.00, 43.00) | 20.00 (4.00, 90.00) | 20.00 (4.00, 77.00) | 13.00 (3.00, 55.00) | 12.00 (3.00, 50.00) | 9.00 (2.00, 44.00) | 11.00 (2.00, 58.00) | 7.00 (2.00, 41.00) | 28.00 (4.00, 195.00) |
| Infectious Diseases Department | 5.00 (1.50, 13.00) | 8.00 (2.00, 25.00) | 9.00 (3.00, 40.50) | 27.00 (5.00, 96.00) | 21.00 (5.00, 94.00) | 15.00 (4.00, 63.75) | 16.00 (3.00, 68.50) | 14.00 (3.00, 65.50) | 16.50 (3.00, 86.25) | 18.00 (4.00, 79.00) | 14.00 (3.00, 65.75) | 15.00 (3.00, 61.00) | 14.00 (3.00, 64.00) | 9.00 (2.00, 40.00) | 7.00 (2.00, 39.00) | 32.00 (5.00, 193.00) |
| Medical Technology Department | 3.00 (1.00, 5.00) | 3.00 (1.00, 10.50) | 5.00 (1.00, 30.50) | 11.00 (3.00, 54.50) | 12.00 (3.00, 48.00) | 8.50 (2.00, 42.00) | 8.00 (2.00, 26.00) | 7.00 (2.00, 37.75) | 11.00 (3.00, 61.50) | 10.00 (2.00, 51.00) | 8.00 (2.00, 37.25) | 9.00 (3.00, 39.00) | 6.00 (2.00, 30.00) | 7.00 (2.00, 39.00) | 5.00 (1.00, 22.00) | 10.00 (2.00, 72.25) |
| Neurology Department | 3.00 (1.00, 9.50) | 6.00 (2.00, 26.00) | 10.00 (2.00, 39.50) | 16.00 (3.00, 71.00) | 18.00 (4.00, 83.50) | 13.00 (2.75, 54.00) | 9.00 (2.00, 40.00) | 8.00 (2.00, 39.50) | 13.00 (3.00, 61.00) | 16.00 (4.00, 62.00) | 10.00 (3.00, 43.00) | 10.00 (2.50, 40.00) | 8.00 (2.00, 38.00) | 8.00 (2.00, 41.00) | 6.00 (2.00, 31.00) | 19.00 (4.00, 136.00) |
| Obstetrics and Gynecology Department | 5.00 (2.00, 18.00) | 9.00 (3.00, 36.00) | 13.00 (4.00, 59.25) | 33.00 (5.00, 138.00) | 35.50 (6.00, 173.00) | 23.00 (4.00, 107.00) | 17.00 (3.00, 106.00) | 25.00 (4.00, 116.00) | 45.50 (7.00, 234.75) | 45.00 (10.00, 168.00) | 29.00 (7.00, 109.00) | 24.00 (5.00, 90.00) | 20.00 (5.00, 84.00) | 16.00 (4.00, 72.00) | 11.00 (3.00, 58.00) | 70.00 (10.00, 462.00) |
| Oncology Department | 3.00 (1.00, 8.00) | 7.00 (2.00, 22.00) | 9.00 (3.00, 33.00) | 9.00 (3.00, 45.00) | 8.00 (2.00, 35.00) | 8.00 (2.00, 29.00) | 6.00 (2.00, 30.00) | 7.00 (2.00, 33.00) | 8.00 (2.00, 36.00) | 13.00 (3.00, 50.00) | 9.00 (2.00, 40.75) | 9.00 (2.00, 39.00) | 6.00 (2.00, 29.00) | 6.00 (2.00, 30.25) | 4.00 (1.00, 22.75) | 13.00 (3.00, 81.50) |
| Orthopedics Department | 4.00 (2.00, 10.00) | 9.00 (2.00, 35.00) | 11.00 (3.00, 56.00) | 19.00 (3.00, 83.00) | 18.00 (4.00, 89.00) | 13.00 (3.00, 55.00) | 9.00 (2.00, 44.00) | 10.00 (2.00, 46.00) | 20.00 (4.00, 96.00) | 20.00 (4.00, 80.00) | 13.00 (3.00, 50.00) | 11.00 (3.00, 46.00) | 9.00 (2.00, 37.00) | 8.00 (2.00, 38.00) | 6.00 (2.00, 26.00) | 26.00 (5.00, 179.00) |
| Otorhinolaryngology Department | 4.00 (1.00, 11.00) | 8.00 (3.00, 29.00) | 11.00 (4.00, 37.50) | 20.00 (5.00, 88.00) | 18.00 (5.00, 99.00) | 15.00 (4.00, 63.00) | 9.00 (2.00, 44.00) | 11.00 (3.00, 48.00) | 21.00 (4.00, 108.00) | 26.00 (6.00, 95.00) | 16.00 (4.00, 63.00) | 13.00 (3.00, 55.00) | 11.00 (3.00, 45.00) | 9.00 (2.00, 44.00) | 6.00 (2.00, 33.00) | 34.00 (6.00, 223.25) |
| Pediatrics Department | 4.50 (2.00, 18.50) | 10.00 (3.00, 45.00) | 12.00 (3.00, 64.25) | 27.00 (5.00, 132.00) | 31.00 (5.00, 141.50) | 20.00 (5.00, 90.25) | 12.00 (3.00, 69.00) | 18.00 (4.00, 79.00) | 42.00 (7.00, 187.00) | 36.00 (8.00, 125.50) | 24.00 (6.00, 89.00) | 22.00 (5.00, 81.00) | 20.00 (4.00, 81.25) | 20.00 (4.00, 87.00) | 15.00 (3.00, 79.00) | 73.00 (10.00, 450.00) |
| Plastic and Cosmetic Surgery Department | 4.00 (2.00, 7.00) | 7.00 (2.00, 30.25) | 18.50 (3.00, 73.25) | 39.00 (6.00, 147.00) | 37.50 (5.00, 149.00) | 25.50 (4.00, 119.75) | 26.00 (4.00, 127.50) | 26.50 (5.00, 111.75) | 59.00 (10.00, 254.00) | 64.00 (11.00, 170.00) | 30.00 (8.00, 93.00) | 27.00 (7.00, 89.00) | 23.00 (6.00, 80.00) | 18.00 (4.00, 70.00) | 12.00 (3.00, 46.00) | 80.00 (12.00, 464.25) |
| Psychiatry and Psychology Department | 6.00 (1.00, 15.00) | 8.00 (3.00, 27.00) | 10.00 (3.00, 40.00) | 22.00 (4.00, 108.00) | 23.00 (5.00, 110.50) | 15.00 (3.00, 66.00) | 9.50 (2.00, 47.50) | 11.00 (2.00, 43.50) | 17.00 (4.00, 111.00) | 24.00 (5.00, 94.25) | 15.00 (3.00, 61.00) | 14.00 (4.00, 59.00) | 13.00 (3.00, 54.00) | 12.00 (3.00, 46.00) | 8.00 (2.00, 34.00) | 34.00 (6.00, 229.50) |
| Pulmonology Department | 5.00 (3.00, 11.25) | 6.00 (2.00, 20.25) | 11.00 (3.00, 31.00) | 15.50 (4.00, 88.50) | 16.00 (3.75, 114.25) | 9.00 (3.00, 60.25) | 12.00 (3.00, 53.00) | 12.00 (2.50, 46.50) | 10.00 (2.00, 65.25) | 15.00 (3.00, 64.00) | 8.00 (2.00, 42.00) | 10.00 (3.00, 39.00) | 9.00 (3.00, 44.00) | 6.00 (2.00, 23.00) | 5.00 (2.00, 28.00) | 17.00 (3.00, 109.00) |
| Traditional Chinese Medicine Department | 4.00 (1.00, 10.00) | 9.00 (3.00, 31.00) | 10.00 (3.00, 45.00) | 21.00 (4.00, 96.00) | 24.00 (5.00, 110.00) | 17.00 (4.00, 67.25) | 10.00 (2.00, 54.00) | 11.00 (3.00, 52.00) | 18.00 (3.00, 103.00) | 22.00 (5.00, 87.00) | 12.00 (3.00, 48.00) | 9.00 (3.00, 36.00) | 8.00 (2.00, 33.00) | 6.00 (2.00, 27.00) | 5.00 (2.00, 19.00) | 22.00 (4.00, 156.00) |
| Urology Department | 3.00 (2.00, 9.75) | 7.00 (2.00, 21.00) | 10.00 (3.00, 44.00) | 19.00 (4.00, 89.00) | 18.00 (4.00, 84.00) | 13.00 (3.00, 49.00) | 9.00 (2.00, 40.75) | 10.00 (2.00, 46.50) | 24.00 (5.00, 110.25) | 29.50 (6.00, 108.25) | 19.00 (4.00, 70.00) | 15.00 (4.00, 61.00) | 13.00 (3.00, 54.00) | 10.00 (3.00, 49.00) | 7.00 (2.00, 36.00) | 39.00 (7.00, 258.00) |
| Other Department | 3.00 (1.00, 8.00) | 5.00 (2.00, 20.00) | 6.00 (2.00, 29.00) | 12.00 (3.00, 59.00) | 11.00 (3.00, 64.00) | 8.00 (2.00, 35.00) | 7.00 (2.00, 37.50) | 7.00 (2.00, 35.00) | 9.00 (2.00, 46.00) | 10.00 (2.00, 45.00) | 7.00 (2.00, 28.00) | 6.00 (2.00, 27.00) | 6.00 (2.00, 29.00) | 6.00 (2.00, 25.00) | 4.00 (1.00, 17.00) | 10.00 (2.00, 63.00) |
| Total | 4.00 (1.00, 11.00) | 7.00 (2.00, 28.00) | 10.00 (3.00, 43.00) | 19.00 (4.00, 87.00) | 19.00 (4.00, 96.00) | 14.00 (3.00, 61.00) | 10.00 (2.00, 51.00) | 12.00 (3.00, 55.00) | 20.00 (4.00, 108.00) | 23.00 (5.00, 92.00) | 15.00 (3.00, 61.00) | 13.00 (3.00, 54.00) | 11.00 (3.00, 50.00) | 10.00 (2.00, 48.00) | 7.00 (2.00, 36.00) | 29.00 (5.00, 203.00) |

### Table S7: The consultation price from 2008 to 2022

| **Group** | **Consultation price from 2008 to 2022, *median* (*IQR*) RMB** | | | | | | | | | | | | | | | |
| --- | --- | --- | --- | --- | --- | --- | --- | --- | --- | --- | --- | --- | --- | --- | --- | --- |
|  | **2008** | **2009** | **2010** | **2011** | **2012** | **2013** | **2014** | **2015** | **2016** | **2017** | **2018** | **2019** | **2020** | **2021** | **2022** | **Total** |
| **Professional title** |  |  |  |  |  |  |  |  |  |  |  |  |  |  |  |  |
| Primary title | 9.00 (9.00, 9.00) | 9.00 (9.00, 9.00) | 9.00 (9.00, 19.00) | 9.00 (9.00, 120.00) | 9.00 (9.00, 19.00) | 9.00 (9.00, 19.00) | 9.00 (9.00, 21.00) | 9.00 (9.00, 19.00) | 9.00 (9.00, 19.00) | 10.00 (9.00, 25.00) | 19.00 (9.00, 30.00) | 11.50 (0.00, 30.00) | 0.00 (0.00, 9.00) | 11.50 (0.00, 37.50) | 10.00 (0.00, 39.00) | 9.00 (0.00, 20.00) |
| Intermediate title | 9.00 (9.00, 15.00) | 9.00 (9.00, 18.00) | 9.00 (9.00, 30.00) | 9.00 (9.00, 30.00) | 9.00 (9.00, 30.00) | 10.00 (9.00, 30.00) | 15.00 (9.00, 39.00) | 12.50 (9.00, 30.00) | 15.00 (9.00, 30.00) | 29.00 (9.00, 50.00) | 30.00 (18.00, 60.00) | 35.00 (11.50, 68.00) | 9.00 (0.00, 40.00) | 37.50 (15.00, 75.00) | 39.00 (17.00, 75.00) | 29.00 (9.00, 50.00) |
| Senior title | 30.00 (9.00, 100.00) | 30.00 (9.00, 60.00) | 30.00 (9.00, 60.00) | 30.00 (9.00, 70.00) | 30.00 (9.00, 80.00) | 30.00 (9.00, 80.00) | 39.00 (15.00, 100.00) | 49.00 (16.00, 100.00) | 35.00 (18.00, 88.00) | 50.00 (25.00, 100.00) | 60.00 (30.00, 119.00) | 69.00 (30.00, 149.00) | 50.00 (15.00, 112.50) | 90.00 (39.00, 159.00) | 99.00 (40.00, 189.00) | 50.00 (25.00, 100.00) |
| **Province** |  |  |  |  |  |  |  |  |  |  |  |  |  |  |  |  |
| Anhui | 9.00 (9.00, 20.00) | 9.00 (9.00, 20.00) | 20.00 (9.00, 40.00) | 20.00 (9.00, 49.00) | 15.00 (9.00, 30.00) | 20.00 (9.00, 39.00) | 20.00 (9.00, 39.00) | 15.00 (9.00, 30.00) | 20.00 (9.00, 30.00) | 30.00 (14.00, 50.00) | 30.00 (20.00, 60.00) | 37.50 (19.00, 69.00) | 19.00 (0.00, 47.50) | 37.50 (20.00, 73.00) | 38.00 (18.00, 80.00) | 29.00 (9.00, 50.00) |
| Beijing | 60.00 (30.00, 150.00) | 60.00 (24.00, 150.00) | 50.00 (15.00, 120.00) | 60.00 (19.00, 150.00) | 60.00 (30.00, 157.00) | 75.00 (30.00, 200.00) | 99.00 (30.00, 200.00) | 100.00 (30.00, 200.00) | 60.00 (30.00, 150.00) | 99.00 (30.00, 200.00) | 100.00 (50.00, 200.00) | 100.00 (50.00, 200.00) | 100.00 (50.00, 200.00) | 150.00 (69.00, 260.00) | 150.00 (75.00, 288.00) | 100.00 (50.00, 200.00) |
| Chongqing | 10.00 (9.00, 30.00) | 28.00 (9.00, 60.00) | 20.00 (9.00, 60.00) | 30.00 (9.00, 60.00) | 30.00 (9.00, 60.00) | 30.00 (10.00, 60.00) | 30.00 (10.00, 60.00) | 30.00 (15.00, 69.00) | 30.00 (10.00, 60.00) | 40.00 (20.00, 80.00) | 50.00 (20.00, 100.00) | 60.00 (20.00, 99.00) | 25.00 (0.00, 80.00) | 50.00 (20.00, 100.00) | 60.00 (20.00, 100.00) | 37.50 (11.50, 80.00) |
| Fujian | 10.00 (9.00, 50.00) | 15.00 (9.00, 50.00) | 9.00 (9.00, 50.00) | 18.00 (9.00, 50.00) | 30.00 (9.00, 50.00) | 23.00 (9.00, 50.00) | 30.00 (9.00, 59.00) | 40.00 (11.50, 96.00) | 30.00 (11.50, 50.00) | 39.00 (20.00, 66.00) | 45.00 (29.00, 90.00) | 49.00 (15.00, 99.00) | 10.00 (0.00, 50.00) | 50.00 (24.00, 99.00) | 50.00 (25.00, 90.00) | 33.00 (10.00, 66.00) |
| Gansu | 9.00 (9.00, 32.00) | 9.00 (9.00, 30.00) | 29.00 (9.00, 30.00) | 30.00 (10.00, 30.00) | 30.00 (9.00, 30.00) | 9.00 (9.00, 30.00) | 10.00 (9.00, 30.00) | 20.00 (11.50, 30.00) | 20.00 (9.00, 30.00) | 20.00 (9.00, 39.00) | 30.00 (15.00, 50.00) | 30.00 (10.00, 50.00) | 15.00 (0.00, 40.00) | 30.00 (10.00, 59.00) | 30.00 (10.00, 60.00) | 29.00 (9.00, 45.00) |
| Guangdong | 35.00 (9.00, 90.00) | 30.00 (9.00, 60.00) | 34.00 (9.00, 80.00) | 35.00 (9.00, 98.00) | 35.00 (9.00, 99.00) | 35.00 (15.00, 90.00) | 49.00 (19.00, 100.00) | 60.00 (30.00, 100.00) | 39.00 (19.00, 90.00) | 50.00 (30.00, 100.00) | 60.00 (30.00, 100.00) | 60.00 (30.00, 120.00) | 50.00 (9.00, 100.00) | 80.00 (35.00, 125.00) | 80.00 (35.00, 150.00) | 50.00 (26.00, 100.00) |
| Guangxi | 10.00 (9.00, 10.00) | 10.00 (9.00, 30.00) | 9.00 (9.00, 15.00) | 9.00 (9.00, 15.00) | 9.00 (9.00, 20.00) | 9.00 (9.00, 24.50) | 10.00 (9.00, 30.00) | 15.00 (9.00, 38.00) | 19.00 (9.00, 30.00) | 30.00 (10.00, 50.00) | 30.00 (18.00, 50.00) | 34.00 (15.00, 50.00) | 19.00 (0.00, 39.00) | 30.00 (18.00, 50.00) | 30.00 (15.00, 60.00) | 29.00 (9.00, 49.00) |
| Guizhou | 9.00 (9.00, 9.00) | 9.00 (9.00, 15.00) | 9.00 (9.00, 30.00) | 9.00 (9.00, 30.00) | 9.00 (9.00, 15.00) | 28.00 (9.00, 30.00) | 30.00 (9.00, 100.00) | 24.00 (9.00, 30.00) | 24.00 (9.00, 37.50) | 30.00 (10.00, 50.00) | 30.00 (16.00, 50.00) | 30.00 (15.00, 50.00) | 11.50 (0.00, 30.00) | 30.00 (19.00, 50.00) | 30.00 (11.50, 55.00) | 29.00 (9.00, 50.00) |
| Hainan | 9.00 (9.00, 15.00) | 9.00 (9.00, 15.00) | 150.00 (9.00, 150.00) | 15.00 (9.00, 30.00) | 30.00 (9.00, 100.00) | 9.00 (9.00, 9.00) | 9.00 (9.00, 49.00) | 30.00 (9.00, 60.00) | 30.00 (15.00, 49.00) | 35.00 (29.00, 50.00) | 37.00 (20.00, 69.00) | 31.50 (20.00, 60.00) | 11.50 (0.00, 30.00) | 35.00 (25.00, 60.00) | 31.50 (12.00, 60.00) | 30.00 (9.00, 50.00) |
| Hebei | 9.00 (9.00, 14.00) | 9.00 (9.00, 15.00) | 9.00 (9.00, 19.00) | 9.00 (9.00, 15.00) | 9.00 (9.00, 19.00) | 9.00 (9.00, 20.00) | 9.00 (9.00, 30.00) | 9.00 (9.00, 30.00) | 15.00 (9.00, 30.00) | 20.00 (9.00, 35.00) | 30.00 (12.00, 50.00) | 29.00 (0.00, 50.00) | 10.00 (0.00, 30.00) | 29.00 (9.00, 50.00) | 30.00 (9.00, 50.00) | 18.00 (9.00, 35.00) |
| Heilongjiang | 9.00 (9.00, 30.50) | 9.00 (9.00, 25.00) | 15.00 (9.00, 30.00) | 19.00 (9.00, 34.00) | 19.00 (9.00, 34.00) | 20.00 (9.00, 58.00) | 30.00 (9.00, 59.00) | 15.00 (9.00, 30.00) | 20.00 (9.00, 39.00) | 30.00 (15.00, 58.00) | 39.00 (29.00, 70.00) | 40.00 (15.00, 90.00) | 30.00 (0.00, 59.00) | 50.00 (23.00, 75.00) | 49.00 (29.00, 80.00) | 30.00 (9.00, 60.00) |
| Henan | 9.00 (9.00, 30.00) | 15.00 (9.00, 50.00) | 13.00 (9.00, 30.00) | 9.00 (9.00, 30.00) | 13.00 (9.00, 30.00) | 10.00 (9.00, 30.00) | 9.00 (9.00, 30.00) | 15.00 (9.00, 30.00) | 19.00 (9.00, 30.00) | 30.00 (11.50, 50.00) | 36.00 (20.00, 75.00) | 39.00 (12.50, 70.00) | 10.00 (0.00, 39.00) | 35.00 (15.00, 70.00) | 30.00 (11.50, 70.00) | 25.00 (9.00, 50.00) |
| Hubei | 30.00 (9.00, 30.00) | 30.00 (9.00, 59.00) | 30.00 (9.00, 45.00) | 30.00 (9.00, 60.00) | 30.00 (9.00, 60.00) | 30.00 (10.00, 60.00) | 30.00 (9.00, 59.00) | 30.00 (9.00, 50.00) | 29.00 (9.00, 59.00) | 37.50 (15.00, 80.00) | 50.00 (29.00, 100.00) | 50.00 (20.00, 100.00) | 30.00 (0.00, 69.00) | 50.00 (29.00, 100.00) | 60.00 (30.00, 100.00) | 37.50 (15.00, 80.00) |
| Hunan | 45.00 (10.00, 60.00) | 10.00 (9.00, 30.00) | 30.00 (9.00, 60.00) | 30.00 (9.00, 60.00) | 30.00 (9.00, 50.00) | 30.00 (9.00, 50.00) | 30.00 (9.00, 59.00) | 30.00 (9.00, 60.00) | 30.00 (10.00, 75.00) | 50.00 (20.00, 99.00) | 50.00 (30.00, 100.00) | 60.00 (30.00, 100.00) | 30.00 (0.00, 80.00) | 60.00 (30.00, 120.00) | 59.00 (30.00, 120.00) | 48.00 (15.00, 99.00) |
| Jiangsu | 30.00 (9.00, 100.00) | 30.00 (9.00, 60.00) | 30.00 (9.00, 60.00) | 30.00 (9.00, 60.00) | 30.00 (9.00, 60.00) | 30.00 (9.00, 60.00) | 30.00 (9.00, 70.00) | 34.00 (11.50, 70.00) | 30.00 (11.50, 60.00) | 40.00 (19.00, 90.00) | 50.00 (29.00, 100.00) | 50.00 (20.00, 100.00) | 30.00 (0.00, 80.00) | 50.00 (30.00, 100.00) | 60.00 (30.00, 100.00) | 40.00 (15.00, 94.50) |
| Jiangxi | 15.00 (9.00, 50.00) | 9.00 (9.00, 17.25) | 10.00 (9.00, 30.00) | 9.00 (9.00, 30.00) | 10.00 (9.00, 30.00) | 9.00 (9.00, 30.00) | 15.00 (9.00, 30.00) | 9.00 (9.00, 20.00) | 20.00 (9.00, 39.00) | 30.00 (10.00, 50.00) | 30.00 (20.00, 60.00) | 36.00 (15.00, 60.00) | 9.00 (0.00, 40.00) | 39.00 (15.00, 60.00) | 30.00 (12.50, 60.00) | 29.00 (9.00, 50.00) |
| Jilin | 9.00 (9.00, 29.00) | 9.00 (9.00, 29.00) | 15.00 (9.00, 30.00) | 29.00 (9.00, 60.00) | 20.00 (9.00, 60.00) | 15.00 (9.00, 60.00) | 19.00 (10.00, 60.00) | 25.00 (9.00, 34.00) | 19.00 (9.00, 35.00) | 30.00 (11.50, 50.00) | 49.00 (20.00, 98.00) | 49.00 (19.00, 90.00) | 15.00 (0.00, 50.00) | 50.00 (30.00, 98.00) | 51.00 (29.00, 80.00) | 30.00 (9.00, 60.00) |
| Liaoning | 60.00 (9.00, 200.00) | 20.00 (9.00, 60.00) | 30.00 (9.00, 40.00) | 13.00 (9.00, 39.00) | 9.00 (9.00, 39.00) | 10.00 (9.00, 49.00) | 15.00 (9.00, 50.00) | 19.00 (9.00, 50.00) | 15.00 (9.00, 45.00) | 30.00 (15.00, 60.00) | 50.00 (29.00, 80.00) | 50.00 (25.00, 100.00) | 30.00 (0.00, 60.00) | 50.00 (29.00, 100.00) | 59.00 (30.00, 100.00) | 30.00 (9.00, 69.00) |
| Neimenggu | 28.00 (13.75, 28.00) | 9.00 (9.00, 30.00) | 9.00 (9.00, 15.00) | 9.00 (9.00, 9.00) | 9.00 (9.00, 30.00) | 30.00 (9.00, 120.00) | 9.00 (9.00, 30.00) | 9.00 (9.00, 30.00) | 15.00 (9.00, 30.00) | 20.00 (9.00, 40.00) | 30.00 (11.50, 50.00) | 20.00 (0.00, 50.00) | 0.00 (0.00, 25.00) | 30.00 (19.00, 50.00) | 30.00 (19.00, 50.00) | 19.00 (9.00, 37.50) |
| Ningxia | 9.00 (9.00, 9.00) | 9.00 (9.00, 9.00) | 9.00 (9.00, 30.00) | 24.00 (9.00, 24.00) | 24.00 (24.00, 24.00) | 20.00 (9.00, 24.00) | 24.00 (9.00, 30.00) | 30.00 (9.00, 59.00) | 20.00 (9.00, 40.00) | 29.00 (10.00, 38.00) | 29.00 (11.50, 36.00) | 29.00 (10.00, 33.00) | 10.00 (0.00, 30.00) | 30.00 (19.00, 40.00) | 30.00 (15.00, 45.00) | 29.00 (10.00, 37.50) |
| Qinghai | 9.00 (9.00, 9.00) | 15.00 (15.00, 15.00) | 15.00 (9.00, 15.00) | 9.00 (9.00, 9.00) | 9.00 (9.00, 9.00) | 9.00 (9.00, 9.00) | 9.00 (9.00, 10.00) | 9.50 (9.00, 15.00) | 29.00 (9.00, 30.00) | 11.50 (9.00, 30.00) | 11.50 (9.00, 30.00) | 15.00 (10.00, 29.00) | 9.00 (0.00, 15.00) | 9.00 (0.00, 15.00) | 0.00 (0.00, 11.50) | 10.00 (9.00, 29.00) |
| Shaanxi | 30.00 (9.00, 50.00) | 15.00 (9.00, 50.00) | 20.00 (9.00, 31.00) | 29.00 (9.00, 50.00) | 29.00 (9.00, 40.00) | 30.00 (9.00, 60.00) | 30.00 (10.00, 60.00) | 30.00 (10.00, 60.00) | 26.00 (9.00, 50.00) | 35.00 (15.00, 80.00) | 50.00 (30.00, 100.00) | 60.00 (29.00, 100.00) | 30.00 (0.00, 80.00) | 59.00 (30.00, 100.00) | 60.00 (29.00, 100.00) | 39.00 (12.50, 90.00) |
| Shandong | 15.00 (9.00, 30.00) | 9.00 (9.00, 30.00) | 20.00 (9.00, 30.00) | 18.00 (9.00, 49.00) | 19.00 (9.00, 50.00) | 20.00 (9.00, 50.00) | 30.00 (9.00, 59.00) | 15.00 (9.00, 30.00) | 20.00 (9.00, 39.00) | 30.00 (10.00, 50.00) | 30.00 (19.00, 60.00) | 35.00 (11.50, 80.00) | 10.00 (0.00, 39.00) | 39.00 (19.00, 88.00) | 40.00 (19.00, 90.00) | 29.00 (9.00, 50.00) |
| Shanghai | 60.00 (35.00, 100.00) | 38.00 (15.00, 80.00) | 50.00 (21.00, 100.00) | 56.00 (30.00, 108.00) | 60.00 (30.00, 120.00) | 60.00 (30.00, 120.00) | 60.00 (30.00, 128.00) | 60.00 (30.00, 120.00) | 66.00 (30.00, 150.00) | 99.00 (49.00, 180.00) | 100.00 (50.00, 200.00) | 100.00 (50.00, 200.00) | 100.00 (45.00, 200.00) | 100.00 (60.00, 200.00) | 100.00 (60.00, 200.00) | 99.00 (49.00, 199.00) |
| Shanxi | 10.00 (9.00, 50.00) | 9.00 (9.00, 14.00) | 15.00 (9.00, 45.00) | 15.00 (9.00, 30.00) | 26.00 (9.00, 49.00) | 30.00 (9.00, 50.00) | 30.00 (9.00, 48.00) | 9.00 (9.00, 30.00) | 18.00 (9.00, 30.00) | 30.00 (9.00, 50.00) | 38.00 (15.00, 62.50) | 49.00 (15.00, 80.00) | 19.00 (0.00, 50.00) | 39.00 (11.50, 80.00) | 37.50 (0.00, 80.00) | 25.00 (9.00, 50.00) |
| Sichuan | 9.00 (9.00, 30.00) | 9.00 (9.00, 60.00) | 9.00 (9.00, 49.00) | 30.00 (9.00, 50.00) | 30.00 (9.00, 50.00) | 30.00 (9.00, 60.00) | 30.00 (9.00, 60.00) | 30.00 (9.00, 69.00) | 30.00 (11.50, 60.00) | 36.00 (19.00, 80.00) | 50.00 (29.00, 100.00) | 50.00 (25.00, 100.00) | 30.00 (0.00, 80.00) | 59.00 (25.00, 100.00) | 60.00 (30.00, 119.00) | 37.50 (12.50, 90.00) |
| Tianjin | 60.00 (29.00, 99.00) | 30.00 (9.00, 60.00) | 30.00 (9.00, 60.00) | 30.00 (15.00, 60.00) | 30.00 (9.00, 69.00) | 30.00 (10.00, 60.00) | 50.00 (28.00, 99.00) | 30.00 (9.00, 60.00) | 30.00 (15.00, 69.00) | 59.00 (30.00, 100.00) | 62.50 (37.50, 120.00) | 80.00 (37.50, 150.00) | 50.00 (11.50, 100.00) | 60.00 (30.00, 125.00) | 76.25 (40.00, 150.00) | 50.00 (25.00, 100.00) |
| Xinjiang | 9.00 (9.00, 30.00) | 9.00 (9.00, 9.00) | 9.00 (9.00, 9.00) | 9.00 (9.00, 30.00) | 9.00 (9.00, 9.00) | 9.00 (9.00, 35.00) | 9.00 (9.00, 35.00) | 9.00 (9.00, 30.00) | 27.00 (9.00, 50.00) | 30.00 (11.50, 50.00) | 30.00 (10.00, 50.00) | 30.00 (11.50, 60.00) | 11.50 (0.00, 30.00) | 30.00 (10.00, 50.00) | 20.00 (0.00, 40.00) | 20.00 (9.00, 50.00) |
| Xizang |  |  |  |  |  | 9.00 (9.00, 9.00) | 19.50 (14.25, 24.75) |  | 30.00 (9.00, 30.00) | 20.00 (20.00, 50.00) | 60.00 (50.00, 60.00) | 60.00 (11.50, 60.00) | 0.00 (0.00, 11.50) | 30.00 (11.50, 60.00) | 0.00 (0.00, 9.00) | 20.00 (0.00, 50.00) |
| Yunnan | 9.00 (9.00, 9.00) | 9.00 (9.00, 45.00) | 10.00 (9.00, 30.00) | 30.00 (9.00, 90.00) | 20.00 (9.00, 60.00) | 20.00 (9.00, 60.00) | 50.00 (9.00, 70.00) | 30.00 (15.00, 60.00) | 30.00 (18.00, 60.00) | 39.00 (20.00, 60.00) | 45.00 (29.00, 80.00) | 37.50 (11.50, 69.00) | 29.00 (0.00, 60.00) | 44.00 (20.00, 88.00) | 30.00 (15.00, 79.00) | 35.00 (11.50, 69.00) |
| Zhejiang | 25.00 (9.00, 30.00) | 15.00 (9.00, 30.00) | 15.00 (9.00, 50.00) | 20.00 (9.00, 50.00) | 30.00 (9.00, 60.00) | 30.00 (9.00, 60.00) | 30.00 (14.00, 70.00) | 30.00 (15.00, 75.00) | 30.00 (19.00, 69.00) | 49.00 (25.00, 90.00) | 50.00 (30.00, 100.00) | 50.00 (28.00, 100.00) | 30.00 (0.00, 90.00) | 60.00 (30.00, 120.00) | 62.50 (30.00, 120.00) | 50.00 (20.00, 100.00) |
| **Department** |  |  |  |  |  |  |  |  |  |  |  |  |  |  |  |  |
| Cardiology Department | 30.00 (15.00, 60.00) | 30.00 (15.00, 60.00) | 30.00 (10.00, 60.00) | 30.00 (15.00, 79.50) | 30.00 (15.00, 80.00) | 44.00 (15.00, 100.00) | 50.00 (20.00, 100.00) | 40.00 (10.00, 100.00) | 30.00 (10.00, 60.00) | 30.00 (11.50, 80.00) | 49.00 (19.00, 100.00) | 50.00 (15.00, 100.00) | 0.00 (0.00, 50.00) | 79.00 (30.00, 150.00) | 80.00 (30.00, 150.00) | 37.50 (10.00, 100.00) |
| Dermatology and Venereology Department | 50.00 (9.00, 200.00) | 40.00 (10.00, 100.00) | 30.00 (15.00, 60.00) | 30.00 (15.00, 90.00) | 50.00 (15.00, 90.00) | 50.00 (20.00, 90.00) | 50.00 (20.00, 100.00) | 50.00 (20.00, 100.00) | 30.00 (15.00, 60.00) | 45.00 (25.00, 98.00) | 50.00 (30.00, 100.00) | 60.00 (30.00, 118.00) | 45.00 (19.00, 99.00) | 54.00 (30.00, 100.00) | 61.50 (30.00, 128.00) | 50.00 (25.00, 100.00) |
| Endocrinology Department | 47.00 (14.00, 125.00) | 60.00 (9.00, 200.00) | 39.00 (14.00, 100.00) | 39.00 (14.00, 90.00) | 36.00 (15.00, 100.00) | 50.00 (15.00, 120.00) | 50.00 (20.00, 100.00) | 35.00 (10.00, 100.00) | 30.00 (13.00, 99.00) | 50.00 (29.00, 100.00) | 68.00 (30.00, 129.50) | 80.00 (30.00, 150.00) | 11.50 (0.00, 90.00) | 80.00 (30.00, 150.00) | 80.00 (39.00, 150.00) | 50.00 (18.00, 120.00) |
| Family Medicine Department | 10.00 (9.00, 10.00) | 9.00 (9.00, 10.00) | 19.00 (9.00, 19.00) | 9.00 (9.00, 19.00) | 15.00 (9.00, 59.00) | 10.00 (9.00, 42.50) | 9.00 (9.00, 9.00) | 9.00 (9.00, 80.00) | 9.00 (9.00, 11.50) | 10.00 (9.00, 20.00) | 11.50 (9.00, 30.00) | 19.00 (0.00, 50.00) | 0.00 (0.00, 15.00) | 19.00 (0.00, 50.00) | 29.00 (0.00, 50.00) | 9.00 (0.00, 20.00) |
| Gastroenterology Department | 30.00 (9.00, 60.00) | 15.00 (9.00, 50.00) | 30.00 (9.00, 60.00) | 35.00 (9.00, 88.00) | 30.00 (9.00, 60.00) | 30.00 (9.00, 60.00) | 35.00 (13.00, 90.00) | 30.00 (19.00, 90.00) | 30.00 (15.00, 90.00) | 50.00 (25.00, 100.00) | 60.00 (30.00, 100.00) | 60.00 (30.00, 125.00) | 40.00 (0.00, 100.00) | 70.00 (30.00, 150.00) | 80.00 (30.00, 150.00) | 50.00 (20.00, 100.00) |
| General Surgery Department | 50.00 (15.00, 90.00) | 30.00 (9.00, 88.00) | 30.00 (9.00, 60.00) | 30.00 (9.00, 60.00) | 30.00 (9.00, 60.00) | 30.00 (9.00, 75.00) | 49.00 (15.00, 90.00) | 30.00 (9.00, 60.00) | 30.00 (9.00, 60.00) | 38.00 (18.00, 90.00) | 50.00 (29.00, 100.00) | 59.00 (29.00, 120.00) | 50.00 (19.00, 100.00) | 69.00 (30.00, 150.00) | 80.00 (39.00, 200.00) | 50.00 (19.00, 100.00) |
| Infectious Diseases Department | 35.00 (9.00, 35.00) | 35.00 (15.00, 90.00) | 35.00 (9.00, 90.00) | 35.00 (15.00, 90.00) | 30.00 (9.00, 90.00) | 35.00 (9.00, 75.00) | 35.00 (9.00, 70.00) | 60.00 (19.00, 100.00) | 40.00 (15.00, 100.00) | 50.00 (30.00, 100.00) | 60.00 (30.00, 135.00) | 60.00 (30.00, 150.00) | 0.00 (0.00, 60.00) | 60.00 (30.00, 150.00) | 62.50 (31.50, 150.00) | 50.00 (10.00, 100.00) |
| Medical Technology Department | 9.00 (9.00, 35.00) | 9.00 (9.00, 15.00) | 15.00 (9.00, 50.00) | 12.00 (9.00, 60.00) | 15.00 (9.00, 60.00) | 30.00 (9.00, 75.00) | 60.00 (10.00, 100.00) | 40.00 (9.00, 90.00) | 40.00 (19.00, 79.00) | 50.00 (29.00, 100.00) | 60.00 (30.00, 100.00) | 60.00 (30.00, 100.00) | 50.00 (20.00, 100.00) | 62.00 (30.00, 150.00) | 70.00 (30.00, 199.00) | 50.00 (20.00, 100.00) |
| Neurology Department | 35.00 (15.00, 100.00) | 30.00 (9.00, 60.00) | 15.00 (9.00, 50.00) | 30.00 (9.00, 70.00) | 30.00 (10.00, 100.00) | 48.00 (15.00, 100.00) | 60.00 (29.00, 166.00) | 30.00 (9.00, 100.00) | 30.00 (15.00, 88.00) | 50.00 (20.00, 100.00) | 62.50 (30.00, 150.00) | 79.00 (30.00, 150.00) | 30.00 (0.00, 100.00) | 99.00 (39.00, 200.00) | 100.00 (40.00, 200.00) | 50.00 (19.00, 125.00) |
| Obstetrics and Gynecology Department | 29.00 (9.00, 45.00) | 30.00 (9.00, 80.00) | 30.00 (9.00, 90.00) | 30.00 (9.00, 60.00) | 30.00 (9.00, 80.00) | 30.00 (9.00, 80.00) | 30.00 (9.00, 70.00) | 30.00 (11.50, 80.00) | 30.00 (10.00, 50.00) | 38.00 (19.00, 79.00) | 50.00 (30.00, 100.00) | 50.00 (22.50, 100.00) | 40.00 (11.50, 100.00) | 60.00 (30.00, 120.00) | 60.00 (30.00, 136.50) | 40.00 (19.00, 99.00) |
| Oncology Department | 30.00 (9.00, 37.00) | 30.00 (9.00, 58.00) | 20.00 (9.00, 60.00) | 35.00 (9.00, 100.00) | 37.00 (9.00, 100.00) | 37.00 (9.00, 100.00) | 60.00 (15.00, 100.00) | 50.00 (10.00, 100.00) | 50.00 (15.00, 100.00) | 60.00 (30.00, 150.00) | 80.00 (30.00, 175.00) | 99.00 (35.00, 200.00) | 80.00 (30.00, 200.00) | 100.00 (50.00, 200.00) | 100.00 (48.00, 268.00) | 75.00 (30.00, 199.00) |
| Orthopedics Department | 30.00 (9.00, 58.00) | 28.00 (9.00, 50.00) | 30.00 (9.00, 60.00) | 30.00 (9.00, 60.00) | 30.00 (9.00, 60.00) | 30.00 (9.00, 60.00) | 30.00 (9.00, 69.00) | 20.00 (9.00, 59.00) | 25.00 (9.00, 50.00) | 30.00 (15.00, 79.00) | 50.00 (20.00, 100.00) | 50.00 (20.00, 100.00) | 37.50 (9.00, 100.00) | 60.00 (30.00, 120.00) | 60.00 (29.00, 128.00) | 35.00 (10.00, 99.00) |
| Otorhinolaryngology Department | 32.00 (9.00, 100.00) | 30.00 (9.00, 60.00) | 30.00 (10.00, 60.00) | 30.00 (9.00, 60.00) | 30.00 (9.00, 60.00) | 30.00 (10.00, 60.00) | 30.00 (15.00, 68.00) | 30.00 (9.00, 60.00) | 25.00 (9.00, 50.00) | 30.00 (14.00, 79.00) | 50.00 (25.00, 100.00) | 50.00 (25.00, 100.00) | 37.50 (9.00, 100.00) | 69.00 (30.00, 125.00) | 60.00 (30.00, 149.00) | 39.00 (12.50, 100.00) |
| Pediatrics Department | 50.00 (20.00, 142.50) | 39.00 (15.00, 100.00) | 39.00 (15.00, 100.00) | 45.00 (15.00, 100.00) | 48.00 (15.00, 100.00) | 60.00 (20.00, 100.00) | 60.00 (30.00, 150.00) | 59.00 (25.00, 100.00) | 39.00 (19.00, 90.00) | 50.00 (25.00, 100.00) | 60.00 (30.00, 120.00) | 80.00 (39.00, 130.00) | 50.00 (9.00, 100.00) | 89.00 (45.00, 150.00) | 90.00 (45.00, 150.00) | 60.00 (30.00, 119.00) |
| Plastic and Cosmetic Surgery Department | 18.00 (9.00, 80.00) | 29.00 (10.00, 79.00) | 29.00 (10.00, 35.00) | 30.00 (10.00, 60.00) | 30.00 (10.00, 60.00) | 30.00 (15.00, 60.00) | 34.00 (20.00, 68.00) | 34.00 (18.00, 60.00) | 30.00 (15.00, 60.00) | 30.00 (20.00, 70.00) | 37.50 (20.00, 80.00) | 49.00 (11.00, 94.00) | 39.00 (9.00, 90.00) | 50.00 (20.00, 100.00) | 60.00 (25.00, 100.00) | 37.50 (15.00, 90.00) |
| Psychiatry and Psychology Department | 100.00 (20.00, 225.00) | 35.00 (20.00, 200.00) | 90.00 (30.00, 200.00) | 80.00 (30.00, 180.00) | 60.00 (30.00, 120.00) | 60.00 (30.00, 149.00) | 79.00 (30.00, 200.00) | 60.00 (30.00, 150.00) | 60.00 (30.00, 117.00) | 80.00 (37.50, 168.00) | 100.00 (50.00, 200.00) | 100.00 (35.00, 200.00) | 60.00 (10.00, 159.00) | 100.00 (30.00, 200.00) | 104.50 (30.00, 299.00) | 85.00 (30.00, 200.00) |
| Pulmonology Department | 28.00 (9.00, 55.00) | 30.00 (9.00, 35.00) | 30.00 (9.00, 40.00) | 30.00 (9.00, 150.00) | 30.00 (9.00, 60.00) | 30.00 (9.00, 60.00) | 30.00 (9.00, 100.00) | 30.00 (9.00, 60.00) | 30.00 (10.00, 79.00) | 49.00 (29.00, 120.00) | 50.00 (30.00, 120.00) | 50.00 (20.00, 139.00) | 0.00 (0.00, 48.00) | 69.00 (29.00, 200.00) | 80.00 (30.00, 200.00) | 35.00 (9.00, 100.00) |
| Traditional Chinese Medicine Department | 20.00 (9.00, 100.00) | 10.00 (9.00, 60.00) | 9.00 (9.00, 50.00) | 9.00 (9.00, 50.00) | 15.00 (9.00, 59.00) | 18.00 (9.00, 50.00) | 25.00 (9.00, 60.00) | 19.00 (9.00, 59.00) | 20.00 (9.00, 50.00) | 30.00 (10.00, 79.00) | 39.00 (19.00, 100.00) | 45.00 (10.00, 100.00) | 15.00 (0.00, 50.00) | 50.00 (15.00, 100.00) | 50.00 (15.00, 100.00) | 30.00 (9.00, 70.00) |
| Urology Department | 35.00 (9.00, 99.00) | 30.00 (9.00, 60.00) | 30.00 (9.00, 80.00) | 30.00 (9.00, 75.00) | 30.00 (15.00, 80.00) | 49.00 (19.00, 78.75) | 47.00 (15.00, 80.00) | 30.00 (15.00, 100.00) | 30.00 (15.00, 60.00) | 48.00 (25.00, 100.00) | 59.00 (30.00, 100.00) | 59.00 (29.00, 100.00) | 50.00 (20.00, 100.00) | 66.00 (30.00, 125.00) | 75.00 (30.00, 130.00) | 50.00 (22.50, 100.00) |
| Other Department | 9.00 (9.00, 45.00) | 20.00 (9.00, 60.00) | 15.00 (9.00, 50.00) | 29.00 (9.00, 80.00) | 30.00 (9.00, 66.00) | 30.00 (9.00, 60.00) | 45.00 (9.00, 100.00) | 19.00 (9.00, 60.00) | 20.00 (9.00, 50.00) | 45.00 (15.00, 100.00) | 50.00 (25.00, 100.00) | 60.00 (25.00, 120.00) | 0.00 (0.00, 50.00) | 80.00 (30.00, 180.00) | 80.00 (30.00, 200.00) | 35.00 (9.00, 100.00) |
| Total | 30.00 (9.00, 90.00) | 30.00 (9.00, 60.00) | 30.00 (9.00, 60.00) | 30.00 (9.00, 60.00) | 30.00 (9.00, 75.00) | 30.00 (9.00, 80.00) | 35.00 (10.00, 99.00) | 30.00 (9.00, 80.00) | 30.00 (9.00, 60.00) | 39.00 (19.00, 96.00) | 50.00 (29.00, 100.00) | 60.00 (29.00, 112.50) | 30.00 (0.00, 99.00) | 68.00 (30.00, 150.00) | 75.00 (30.00, 150.00) | 49.00 (15.00, 100.00) |
